# Comparative efficacy and acceptability of anxiolytic drugs for the treatment of anxiety disorders: a systematic review and network meta-analysis

**DOI:** 10.1007/s00406-025-02082-0

**Published:** 2025-08-11

**Authors:** Thomas J. Müller, Arnaud Künzi, Ellen Heitlinger, Bernd Krämer, Roland von Känel, Josef Hättenschwiler, Matthias Hilpert, Christian Imboden, Edith Holsboer-Trachsler, Martin Hatzinger, Siegfried Kasper, Borwin Bandelow, Erich Seifritz

**Affiliations:** 1 Private Clinic Meiringen, Meiringen, 3860 Switzerland; 2 University Hospital of Psychiatry and Psychotherapy, University of Bern, Bern, Switzerland; 3 Department of Clinical Research, University of Bern, Bern, Switzerland; 4 H+O communications Ltd, Zurich, 8041 Switzerland; 5 Section for Experimental Psychopathology and Neuroimaging, Department of General Psychiatry, Heidelberg University Hospital, Heidelberg, Germany; 6 Centre for Translational Research in Systems Neuroscience and Psychiatry, Department of Psychiatry and Psychotherapy, University Medical Center Göttingen, Göttingen, Germany; 7 Department of Consultation-Liaison-Psychiatry and Psychosomatic Medicine, University Hospital Zurich, University of Zurich, Zurich, Switzerland; 8 Centre for Anxiety and Depression Treatment Zurich, Zurich, Switzerland; 9 Psychiatric Services of the Canton Aargau, Windisch, Switzerland; 10 Private Clinic Wyss, Münchenbuchsee, Switzerland; 11 Gesundheitszentrum St. Johann, University of Basel, Basel, Switzerland; 12 Psychiatric Services Solothurn and University of Basel, Solothurn, Switzerland; 13 Department of Psychiatry and Psychotherapy, Medical University of Vienna, Vienna, Austria; 14 Comprehensive Center for Clinical Neurosciences and Mental Health, Medical University of Vienna, Vienna, Austria; 15 Center for Brain Research, Medical University of Vienna, Vienna, Austria; 16 Department of Psychiatry and Psychotherapy, University Medical Center Göttingen, Göttingen, Germany; 17 Department of Adult Psychiatry and Psychotherapy, Psychiatric Hospital, University of Zurich, Zurich, Switzerland

**Keywords:** Generalized anxiety disorder, GAD, Anxiety disorders, Silexan, Network meta-analysis, Systematic review

## Abstract

**Background:**

Anti-anxiety medications’ side effects and dependency risks necessitate thorough consideration of their efficacy and acceptability when making treatment decisions.

**Methods:**

A systematic review and Network Meta-Analysis (NMA) was conducted using three databases from 1980 to 2020, focusing on adults diagnosed with generalized anxiety disorder (GAD) or any relevant diagnostic classification of anxiety disorder or those for whom Hamilton Anxiety (HAM-A) data is available, and including any comparator (placebo and/or active comparator treatments). The primary outcomes were efficacy (mean difference in change from baseline in HAM-A total score) and acceptability (study discontinuations for any cause). This study is registered with Open Science Framework (OSF) database.

**Findings:**

Analysis of 100 trials involving 28,637 participants showed that most active drugs were more effective than placebo in reducing anxiety. Notably, silexan was the only phytopharmaceutical included in the NMA. Clomipramine had the highest efficacy and vortioxetine the least. However, in terms of acceptability, clomipramine led to the most study discontinuations, while clobazam had the lowest discontinuation rate, indicating that efficacy and acceptability do not always align. Notably, silexan was both highly effective and as acceptable as a placebo. Only four treatments showed fewer adverse events than placebo (diazepam, agomelatine, clobazam, and silexan).

**Conclusion:**

This network meta-analysis provides a unique comparison of the efficacy and acceptability of anxiolytics. It represents one of the most comprehensive evidence bases available to guide the first choice of treatment for anxiety disorders in adults.

**Supplementary Information:**

The online version contains supplementary material available at 10.1007/s00406-025-02082-0.

## Introduction

Generalized anxiety disorder (GAD) is among the most common forms of anxiety disorder [1–3]. This debilitating illness, characterized by continuous, excessive and unrealistic worry about day-to-day events and problems, exhibits an annual prevalence rate ranging from 0.2 to 4.3% [1, 3]. However, a significant proportion of cases remain undiagnosed or undertreated [1, 4]. Women are more likely to suffer from GAD throughout their lives compared to men, with a female-to-male ratio ranging from 1.7 to 2.6 [3]. GAD is often associated with other anxiety and somatoform disorders, as well as with major depression [5, 6]. Even in the absence of comorbidity, GAD can lead to substantial disability, imposing a considerable personal, societal, and economic burden [6]. Without treatment, GAD typically follows a chronic path with few complete remissions, and most patients continue to experience symptoms six to twelve years after the initial diagnosis [5, 7]. Recent years have seen stricter diagnostic criteria for GAD, which now excludes more patients with significant anxiety who do not meet certain diagnostic criteria from receiving effective treatment [8, 9]. These patients, often labeled as having subclinical or anxiety disorder not otherwise specified, bear a similar disease burden to those diagnosed with full GAD and are frequently at risk of progressing into full GAD or other anxiety disorders [10–12].

Current treatments for anxiety disorders such as GAD might incorporate psychotherapy, anxiolytic drugs, or a combination thereof [1, 13, 14]. Personalized, high-intensity psychological intervention, such as cognitive behavioral therapy (CBT) or applied relaxation techniques, is the most common psychological strategy for treating individuals with GAD who warrant treatment [14, 15]. Antidepressants, particularly selective serotonin reuptake inhibitors (SSRIs) and serotonin-norepinephrine reuptake inhibitors (SNRIs), are recommended pharmacological treatments for GAD and are often the first choice due to their effectiveness and tolerability in many patients [1, 13, 14]. Other classes of anxiolytic drugs may be considered in some patients, such as those with severe GAD, inadequate response or those who have not responded adequately to first-line treatments. Evidence-based alternative anxiolytic drugs that have a different mechanism of action (e.g., anticonvulsants, tricyclic agents, benzodiazepines, atypical antipsychotics) may be considered on- or off-label [1, 13, 14]. However, some anti-anxiety drugs such as benzodiazepines (e.g., lorazepam) and tricyclics (e.g., buspirone) are contraindicated for first-line GAD treatment because of side effects, limited data supporting their use as initial monotherapy, and/or the risk of benzodiazepine dependence associated with chronic use [1, 14]. Additional treatment strategies for anxiety disorders are therefore warranted.

Patients’ preferences and acceptability of anti-anxiety treatments can lead to improved outcomes for both patients and healthcare providers [16]. A meta-analysis noted that patients with psychiatric disorders (mainly depressive or anxiety disorders) have a three-fold preference for psychological treatment relative to pharmacological treatments, likely due to significant medication side effects, potentially leading to lower patient acceptance [17]. This highlights the need for medicines with a favorable tolerability profile that can be used in real-world practice [17]. Phytomedicines often address this need, but evidence regarding the anxiolytic effects of various, widely used over-the-counter phytomedicines is mostly limited [1, 18–20]. Among these phytomedicines, silexan, an oral lavender oil preparation, stands out due to its robust clinical evidence and good tolerability. Silexan has been evaluated in preclinical and clinical studies for its potential use in treating anxiety disorders, including GAD [21, 22]. In multicenter, double-blind, randomized GAD clinical trials, silexan showed comparable reductions in the Hamilton Rating Scale for Anxiety (HAM-A) total score when compared to the benzodiazepine lorazepam [21] and SSRI paroxetine [22], and it was found to be superior to placebo [22]. A network meta-analysis (NMA) of five randomized control trials examined the anti-anxiety benefits of silexan in comparison to placebo and two active comparators (lorazepam and paroxetine) in patients with an anxiety disorder [23]. Overall, treatment with silexan at the higher effective dose resulted in a more significant decline in the HAM-A score across all the comparators and was considered the most effective anxiolytic intervention of the included studies by the study authors [23]. Furthermore, an extensive systematic review and NMA compared the efficacy and acceptability of 22 active GAD treatments or a placebo [24]. However, this study did not include silexan and was only focused on GAD, but not all types of anxiety disorders [24]. Thus, there has not yet been a comprehensive NMA to evaluate the efficacy and acceptability of silexan in comparison with other anxiolytic agents used in the treatment of different anxiety disorders, including GAD.

In this systematic review and NMA, we aimed to build on the evidence from previously conducted NMAs by comparing the efficacy and acceptability evidence of various anxiolytic drugs, including silexan for the first time, as the only included phytomedicine, to determine the most advantageous agents for treating anxiety disorders, including GAD.

## Methods

The systematic review and results of this NMA are reported in accordance with the Preferred Reporting Items for Systematic Reviews and Meta-Analyses (PRISMA-NMA) [25, 26]. They are registered in the Open Science Framework (OSF) database (registration doi: https://osf.io/fyu2j).

### Population, intervention, comparison and outcomes (PICO)

The review focused on the following research question: “What is the efficacy, acceptability and safety profile of silexan compared to other synthetic anxiolytic drugs approved in Switzerland for treating anxiety disorders?” The study was designed using the PICO model as follows: participants (adults ≥ 18 years with a diagnosis of GAD or any relevant anxiety disorder diagnostic classification or Hamilton anxiety rating scale [HAM-A] data), intervention (any placebo-controlled study of drugs), comparator (head-to-head studies of drugs), primary efficacy (change from baseline in a standardized observer-rating scale for anxiety, i.e., HAM-A total score at baseline and the end of the treatment period) AND acceptability (quantified as discontinuation of randomized treatment due to any cause) outcomes. Secondary outcomes (percentage of participants being responders [HAM-A decrease ≥ 50% between baseline and end of treatment], percentage of participants achieving remission [HAM-A < 10 at the end of treatment], quality of life parameters, somatic anxiety factor scores and clinical global impression) could not be analyzed due to a lack of reporting and were not further investigated. Safety outcomes were analyzed as additional secondary outcomes (discontinuations of treatment due to patients experiencing an adverse event, total number of adverse events (AEs) and AE severity).

### Search strategies and study selection

The meta-analysis involved a systematic search of PubMed, Cochrane Library and PsychoINFO databases from 01 January 1980 to 23 March 2020. The search strategies for clinical trials comprised the following relevant drug subject heading terms without any restriction of countries or languages in the title/abstract words: (5-hydroxytryptophan; agomelatine; alpidem; alprazolam; bromazepam; bupropion; buspirone; citalopram; clobazam; clomipramine; desvenlafaxine; diazepam; duloxetine; escitalopram; fluoxetine; gg-HMP; hydroxyzine; imipramine; lorazepam; metaclazepam; opipramol; oxazepam; paroxetine; prazepam; pregabalin; quetiapine; sertraline; silexan; trazodone; venlafaxine; vortioxetine). In addition, the PubMed search strategy also included a study specification term (randomized controlled trial; placebo; drug therapy; random; trial; groups) and an indication (anxiety; generalized anxiety disorder; GAD; or Hamilton anxiety rating scale; HAM-A). The Cochrane database was screened for additional keywords, such as “anxiety disorder” (including word variations), in the title or abstract. The PsychoINFO database was screened for the additional keywords: “anxiety disorder” (randomized OR controlled).

Two reviewers (TM and EH) independently conducted study selection in accordance with pre-specified inclusion criteria, and any disagreements were settled via discussion with the study team. After removing duplicates, the titles and abstracts of remaining records were screened for relevance, the remaining reports were read in full text, and eligible studies were identified. Studies were excluded for the following reasons: (1) non-blinded; (2) where the article could not be obtained; (3) where the study administered two or more medications or two or more premedication phases, i.e., studies that test combination therapies (more than one drug is allocated to one patient); (5) pooled and review studies; (6) studies where no endpoint relevant to our NMA was available; (7) studies with open-label phases other than for wash-out reasons; (8) non-comparable studies due to complex or unusual study design (e.g. cross-over studies, studies including interacting comedications by design like selective serotonin reuptake inhibitor (SSRI)/(serotonin noradrenalin reuptake inhibitor (SNRI)); (9) studies missing a wash-out period for patients already on some study medications; (10) studies with internally contradicting findings and studies with a research aim too remote from the meta-analysis objective; finally (11) multi-arm studies not reporting all comparisons or not reporting all standard errors. Additionally, for multi-arm studies with various doses of the same product, only the arm with the most commonly indicated dosage for the treatment of an anxiety disorder is considered; this determination is based on a pre-specified table established for this purpose (supplementary material Tables S1 and S2). Regarding the studies involving GAD patients, these were included if they included adult patients (≥ 18 years) diagnosed with GAD according to the International Classification of Diseases 9-11th Revision (ICD-9-11) [8], or the American Psychiatric Association’s Diagnostic and Statistical Manual of Mental Disorders (DSM-3-TR4 or DSM-3-TR5) [9], or any other relevant diagnostic classification pre-dating the use of the term ‘GAD’ in the Diagnostic and Statistical Manual of Mental Disorders (DSM-3) in 1980 [27]. 

### Data extraction

Data extraction was performed by three independent reviewers (K AY-Y, JS, and MI). The following data were extracted from each selected study: trial characteristics (publication year, study design, sample size), population characteristics (age, sex, disease severity), intervention characteristics such as duration, nature (fixed or flexible) and dosage of treatment as well as primary efficacy outcomes (efficacy measure according to the HAM-A scale), acceptability (all-cause discontinuation), and safety outcomes (discontinuation due to AEs, number of patients with AEs as totals and per classes: severe [dependency]; moderate [sedation/somnolence, drug-drug interactions, influenza, gastroenteritis, bronchitis, arthralgia]; and light [eructation, headache, nasopharyngitis, diarrhea, nausea, dyspepsia, dizziness]). In addition, it was initially considered to collect data on the Hamilton Depression scale (HAM-D), clinical global impression (CGI), Montgomery Åsberg depression rating scale (MADRS), Pittsburg sleep quality index (PSQI), Sheehan disability scale (SDS) sf36 score, Liebowitz social anxiety scale (LSAS), World Health Organization quality of life (WHOQOL). As described above, shortly after data collection began, those items were discontinued due to a combination of resource limitations, lack of endpoint availability and irrelevance due to the refinement of our research question. Additionally, we excluded the following nine drugs from consideration after the search as they were considered as primarily intended for antidepressant use rather than acting as anti-anxiety drugs: 5-hydroxytryptophan, alpidem, bupropion, desvenlafaxine, gg-HMP, hydroxyzine, imipramine, opipramol and trazodone.

### Data synthesis/imputation

The mean differences in HAM-A total scores between the treatment groups were extracted from the relevant publications. When the standard error (SE) of the mean difference was not reported, it was estimated from the means, the sample sizes and the standard error of the means. When the latter were also not available, the SE was estimated using the mean change or difference in mean change, the inverse normal distribution and in order of availability: the exact p-value, the reported upper bound of the p-value (0.001, 0.01, 0.05, 0.1), and the lower bound of the 95% confidence interval. When the unadjusted arithmetic mean was not reported but rather an adjusted estimate (analysis of variance [ANOVA], analysis of covariance [ANCOVA], linear regression, etc.), the simplification was made to use the adjusted measurements instead.

### Assessment of study quality and risk of bias

The quality of the trials included was assessed in accordance with the Cochrane Collaboration’s tool for assessing the risk of bias (RoB2) as described in the Cochrane Collaboration Handbook [28]. At the network level, two investigators (TM and AK) independently determined the risk of bias using the Confidence in Network Meta-Analysis (CINeMA) framework along five dimensions: within-study bias, indirectness, incoherence, imprecision and heterogeneity [29]. However, the reporting bias dimension could not be investigated due to technical difficulties with the online tool, and it was assessed separately using funnel plots. Consensus with a third investigator (EH) resolved any inconsistency in confidence rating. Incoherence, imprecision, and heterogeneity were investigated and graded on a scale of “no concerns,” “some concerns” and “major concerns” following the CINeMA approach and using a clinically relevant value of 2.5 in difference on the HAM-A scale for the HAM-A endpoint and 0.1 on the relative risk scale for the adverse events endpoint; the subject-matter expert, TM, selected these values based on the magnitude of “very common” of 1/10 of the scale defined by Swissmedic [30]. We refer our readers to Nikolakopoulou A. et al. (2020) [29] for an in-depth explanation of the grading. Beyond the CINeMA criteria, inconsistency was further investigated using forest plots splitting direct and indirect contributions as well as their relative importance to each pairwise network estimate. Heterogeneity/confounding was further tested with forest plots splitting networks at the mean confounding value at the study average level.

### Statistical analyses

The efficacy and safety endpoints were analyzed using a frequentist NMA and a random-effects model. The choice of a random effects model was made given the expected heterogeneity in study designs, disease severity and response to the treatment, inhomogeneous study populations across time, space, differing treatment lengths and dosage. For the HAM-A endpoint, the NMA random effects model was fitted using the changes from baseline rather than the raw values to account for the expected high heterogeneity in the study populations. For all other endpoints (binary outcomes), the denominator chosen was the number of patients randomized. In addition to the total number of AEs, an AE severity endpoint was created by the subject-matter expert, TM, which assigned AEs to one of three severity levels based on the symptom class: light, moderate and severe. Note that for simplification, the assumption is made that patients do not experience more than one type of AE within a severity level. Patients certainly can experience more than one type of AE and usually do, so this assumption is naturally incorrect (albeit necessary due to the non-availability of patient-level data). However, this issue should be relatively independent of whether or not the medication is used to the extent that it would impact the P-score ranking. Results were then obtained using the R package netmeta: First, pairwise differences were generated using the function pairwise with placebo as the reference group. Second, a frequentist random effects NMA was fitted using the netmeta function with argument random set to TRUE. At this stage, single studies that were disconnected from the main network or prevented the fitting of the model due to the unavailability of precise standard error estimates were removed. We finally produced network visualizations, forest plots and diagnostic statistics from the fitted model. The network produced was investigated by use of network graphs to evaluate connectedness. This was done qualitatively by means of a commentary as well as quantitatively (balance of evidence, study characteristics, patient characteristics and diversity between treatments, number of randomized patients per treatment). Extent of heterogeneity and study variability was assessed using the I^2^ and τ metric. Results were then summarized as forest plots depicting point estimates (mean difference for the HAM-A endpoint, risk ratios and differences for binary outcomes), 95% predictive intervals, 95% confidence intervals and p-scores to produce rankings. The NMA estimates were not adjusted on the study and patients’ characteristics. Still, summary statistics were compiled to aid the assessment of their potential impact on the results (supplementary material Tables S5 and S6). All analyses were conducted using R version 4.2 [31] and R package netmeta version 2.7-0 [32]. 

## Results

### Literature selection and study characteristics

**Fig. 1:**
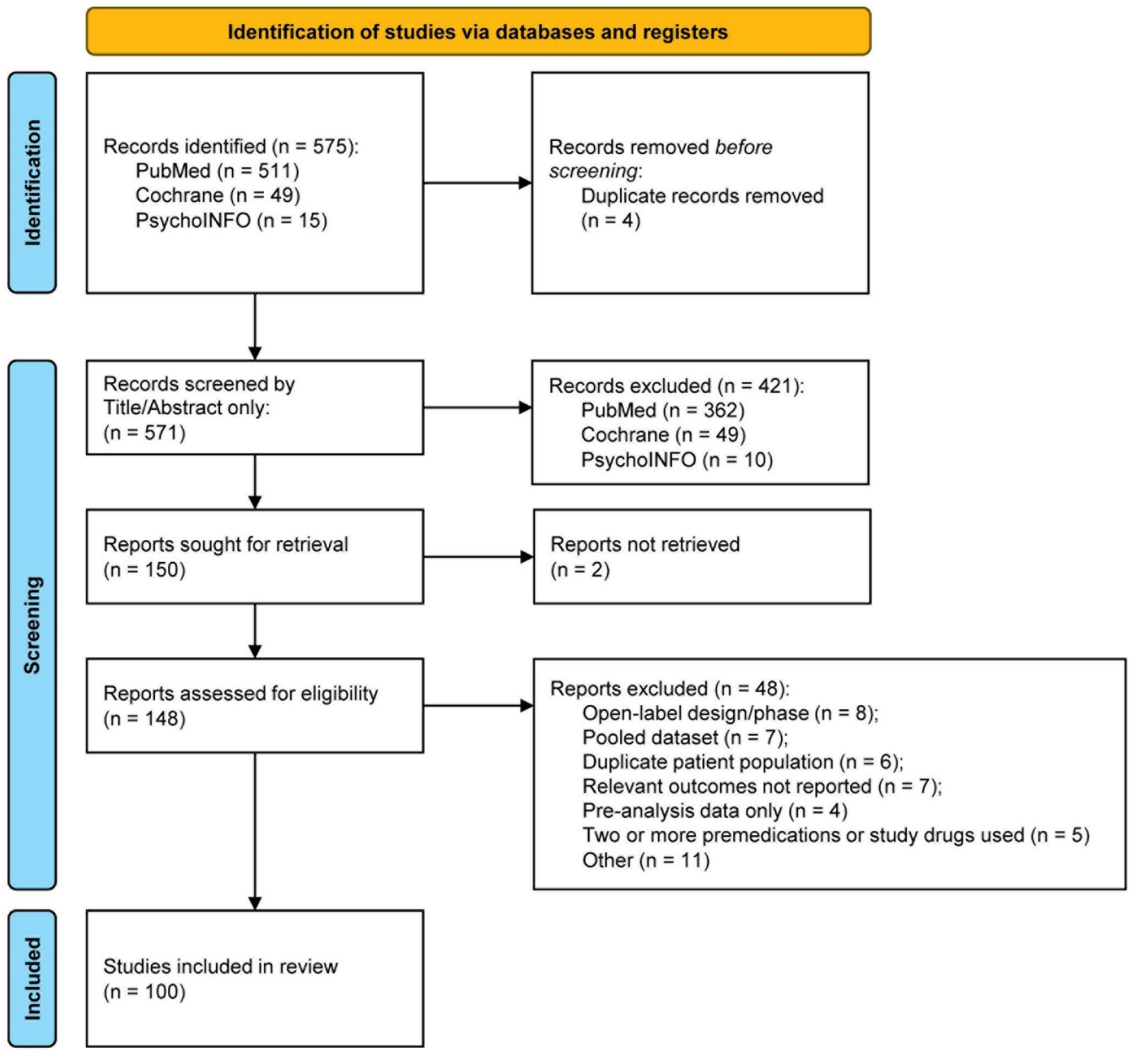
PRISMA flowchart of included studies

The PRISMA flow chart is shown in Fig. 1. After reviewing 575 records from all databases, 100 randomized controlled trials (RCTs) with 28,637 randomized participants met eligibility criteria and were included in this meta-analysis. The mean study length was 9.5 weeks (weighted 10.8 weeks). A higher percentage of studies focused on assessing a flexible drug dosage (68%) compared to those analyzing a fixed dosage (34%), with a small fraction of studies (2%) incorporating both fixed and flexible dosing approaches. The 100 included studies provided efficacy and safety results for 20 treatments (agomelatine, alprazolam, bromazepam, buspirone, clobazam, clomipramine, diazepam, duloxetine, escitalopram, fluoxetine, lorazepam, metaclazepam, oxazepam, paroxetine, pregabalin, quetiapine, sertraline, silexan, venlafaxine, and vortioxetine). The characteristics of the included studies are presented in Table 1 by treatment class and network and in the supplementary material (Tables S3–S6). Of the 100 studies, the following anxiety disorders were included: GAD (*n* = 67 studies), mixed anxiety-depression (MADD) (*n* = 6 studies), GAD with panic disorders (*n* = 2), anxiety disorder not otherwise specified (NOS) (DSM-IV: 300.00; ICD-10: F41.9) (*n* = 1 studies), anxiety disorders (DSM-III) (*n* = 2), social anxiety disorder (SAD) (*n* = 11 studies), anxiety-related restlessness and insomnia (*n* = 1 study), anxiety neurosis (*n* = 5 studies), undefined anxiety disorder (*n* = 5 studies).

### Quality assessment of included studies

**Fig. 2:**
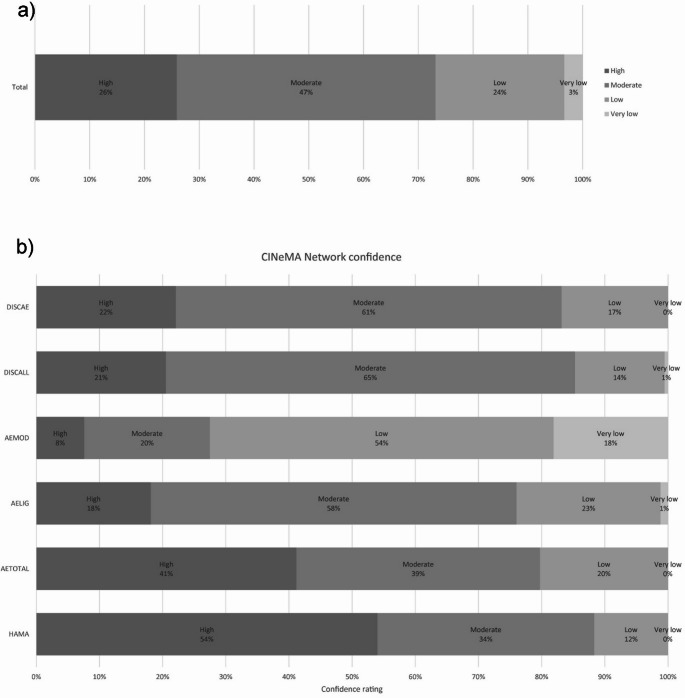
Confidence ratings distribution: **a** overall and **b** per network. *AE* adverse event; *AETOTAL* total AEs; *AELIG* light AEs; *AEMOD* moderate AEs; *DISCALL* all-cause discontinuations; *DISAE* AE-caused discontinuations; *HAM-A* Hamilton Anxiety Rating Scale

The risk of bias graph is shown in Fig. 2a. Of the included 100 studies, 73 studies were graded as having a low risk of bias (high to moderate risk of confidence) and 27 studies were identified as having a high risk of bias. Regarding the domain assessments, HAM-A and total count AEs provided the highest confidence ratings (Fig. 2b; Table 2). In contrast, AE-light and AE-moderate domain assessments provided the lowest confidence ratings (Fig. 2a and b).

**Fig. 3:**
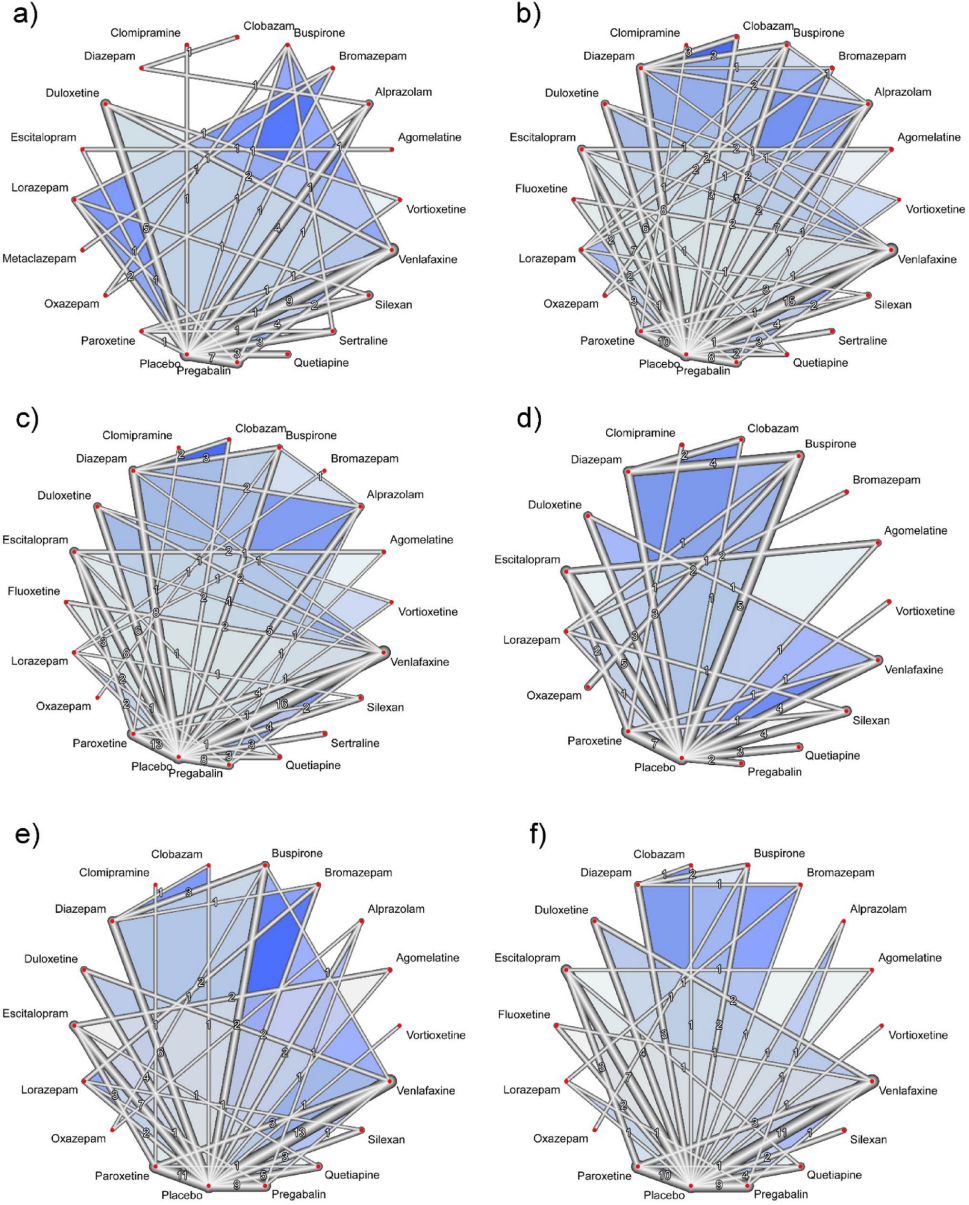
Network diagram for network meta-analysis: **a** Network plot of efficacy (based on HAM-A score); **b** Network plot of acceptability (based on all-cause discontinuations); Network plot of safety based on **c** AE-caused discontinuations; **d** AEs; **e** light AEs; and **f** moderate AEs. The thickness of network lines indicates the number of comparisons between each treatment. Blue shaded area indicates multi-arm studies (> 2 arms). *AEs* adverse events; *HAM-A* Hamilton Anxiety Rating Scale

**Fig. 4:**
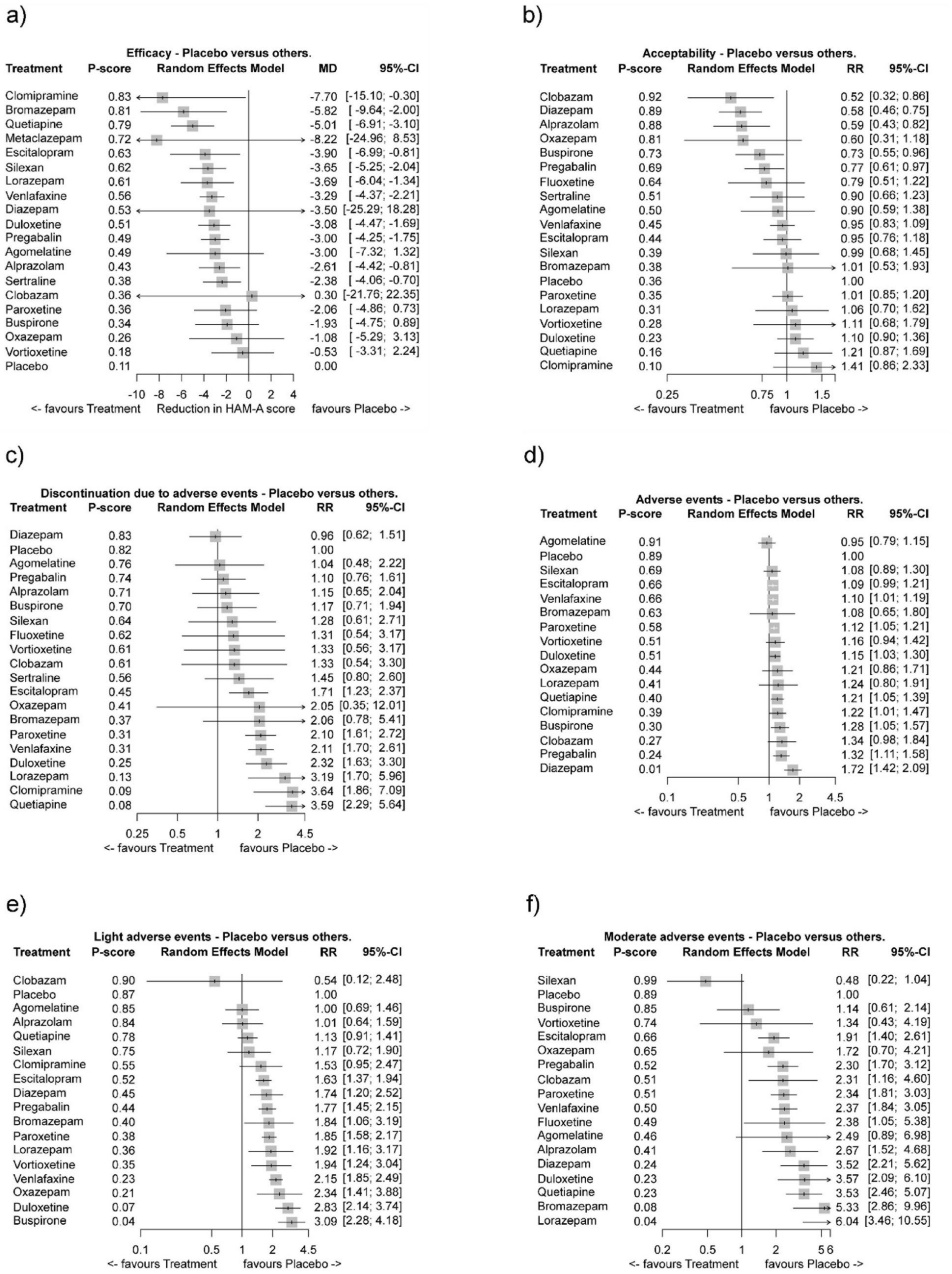
Forest plots with pairwise comparisons of placebo vs. all included treatments for networks: **a** HAM-A, **b** all-cause discontinuations, **c** AE-caused discontinuations, **d** AEs, **e** Light AEs and **f** moderate AEs. *AEs* adverse events; *HAM-A* Hamilton Anxiety Rating Scale

**Fig. 5:**
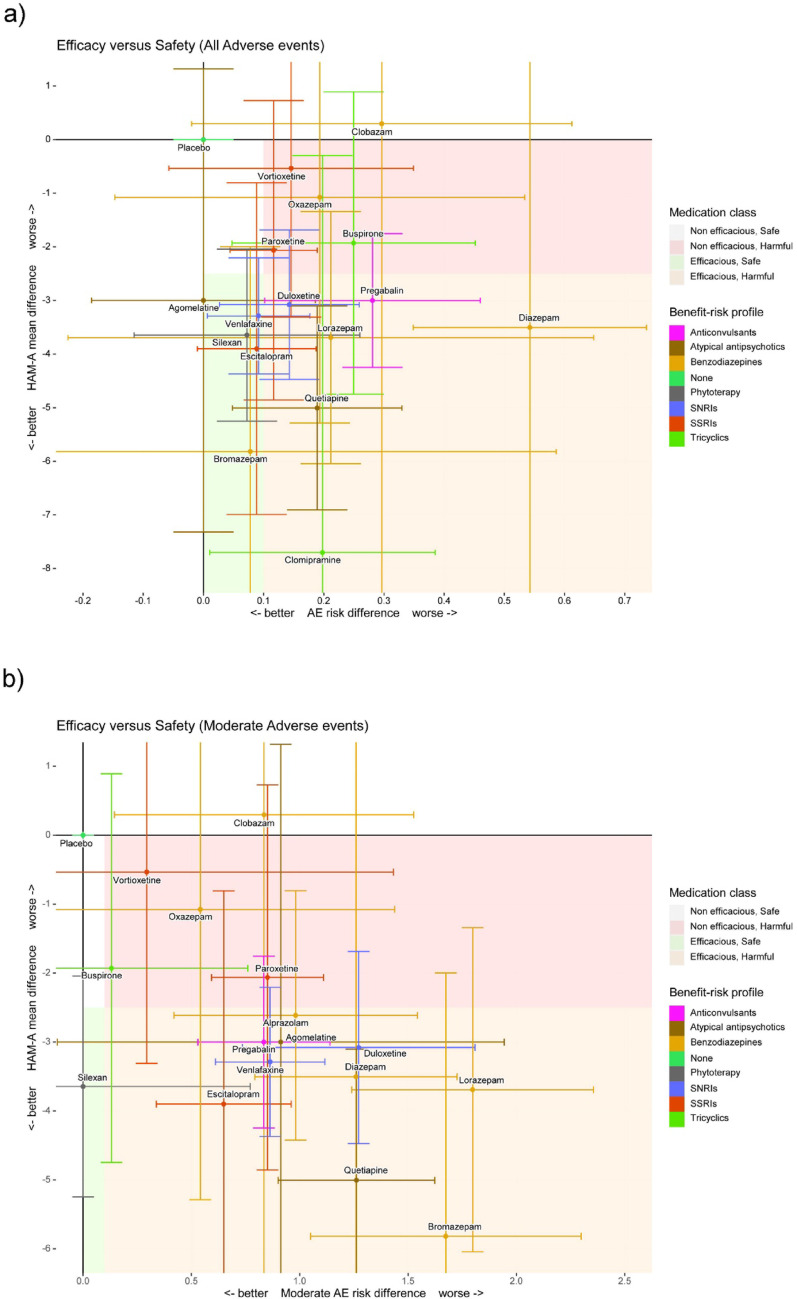
Plot of HAM-A and **a** total AE endpoints or **b** moderate AE endpoints by treatment class. Error bars depict 95% confidence intervals. Grey shaded regions are clinically non-significant and depict the margin within which a treatment is clinically not distinguishable from placebo, i.e., this is the region within which we do not consider the difference to placebo as clinically relevant and correspond to the margin chosen in the CINeMA assessment (i.e., -2.5 on the HAM-A scale and 0.1 in the rate of adverse events). *AE* adverse event; *HAM-A* Hamilton anxiety rating scale. The treatments alprazolam, metaclazepam and sertraline are missing from 5a as they could not be included in the figure due to lack of data availability on both endpoints. The treatments clomipramine, metaclazepam, sertraline and fluoxetine could not be included in 5b for the same reasons

### Evidence network of eligible comparisons

Included and excluded studies based on the network are shown in the supplementary material (Fig. S1). A total of 20 treatment types were analyzed, of which 43 studies were included in the efficacy analysis assessed by HAM-A and 69 studies were included in the acceptability analysis assessed by all-cause discontinuations. Safety outcomes were analyzed in 66 studies assessed by AE discontinuations, 39 studies assessed by AEs, 61 studies assessed by light AEs, and 49 studies assessed by moderate AE.

The networks of the anxiolytic treatments and placebo according to analysis (HAM-A, all-cause discontinuations, AE-caused discontinuations, AEs, light AEs, and moderate AEs) are shown in Fig. 3a–f.

### Efficacy, acceptability and safety outcomes

#### Efficacy of anxiolytic treatments

Forest plots of 19 treatment types showing the mean difference (MD) in HAM-A score are represented in Fig. 4a. Fluoxetine was the only treatment with no HAM-A data and was not included in this analysis. The overall result of the NMA for efficacy favored all included treatments except clobazam compared to placebo, with the highest comparative efficacy demonstrated for clomipramine (MD: -7.70; 95% CI: -15.10 to -0.30) and the lowest comparative efficacy demonstrated for vortioxetine (MD: -0.53; 95% CI: -3.31 to -2.04). In terms of efficacy, using the cutoff value of -2.5MD in HAM-A, as this coincides with a 2.5MD clinically important value, the best treatments were clomipramine, bromazepam, quetiapine, metaclazepam, escitalopram, silexan, lorazepam, venlafaxine, diazepam, duloxetine, pregabalin, agomelatine and alprazolam (Fig. 4a).

#### Acceptability of anxiolytic treatments

All treatments except metaclazepam were included in the meta-analysis for acceptability. Forest plots showing the risk ratio (RR) for all-cause discontinuations revealed that clomipramine, which was the most efficacious treatment by HAM-A score, was less acceptable than placebo and had the highest, although non-significant, reported all-cause risk of discontinuation of the treatments analyzed (RR: 1.41; 95% CI: 0.86 to 2.33). In contrast, treatment with clobazam, deemed the least efficacious treatment versus placebo by HAM-A score, significantly showed the least risk of all-cause discontinuation (RR: 0.52; 95% CI: 0.32 to 0.86). Compared to placebo, clobazam, diazepam, alprazolam, oxazepam, buspirone, pregabalin, fluoxetine, sertraline, agomelatine, venlafaxine and escitalopram all had lower all-cause discontinuations than placebo, with RRs ranging from 0.52 (95% CI: 0.32 to 0.86) for clobazam to 0.95 (95% CI: 0.76–1.18) for escitalopram (Fig. 4b). Four of the six benzodiazepines analyzed had lower all-cause discontinuations than placebo. Two treatments, silexan (RR 0.99 [95% CI: 0.68 to 1.45]) and bromazepam (RR 1.01 [95% CI: 0.53 to 1.93]) were at least as acceptable as placebo based on all-cause discontinuations. The remaining six treatments (paroxetine, lorazepam, vortioxetine, duloxetine, quetiapine and clomipramine) were less acceptable than placebo, with RRs ranging from 1.01 to 1.41.

Eight of the top thirteen most efficacious treatments based on HAM-A score were at least as acceptable as placebo based on all-cause discontinuations (Fig. 5a and b). Notably, silexan was at least as acceptable as placebo in terms of all-cause discontinuations (RR: 0.99; 95% CI: 0.68 to 1.45), indicating a good efficacy/acceptability ratio.

#### Safety of anxiolytic treatments

Overall, only four treatments had a lower risk ratio than placebo in the safety meta-analyses (diazepam had fewer discontinuations due to AEs, agomelatine had fewer AEs, clobazam had fewer light AEs, and silexan had fewer moderate AEs) (Fig. 5c–f). Of these four treatments, only two, silexan and diazepam, were amongst the best thirteen most efficacious treatments based on HAM-A score.

All treatments, except metaclazepam due to limited data, were included in the safety meta-analysis for discontinuations due to AEs (Fig. 4c). Most treatments had more discontinuations due to AEs than placebo, with RRs ranging from 1.04 (95% CI: 0.48 to 2.22) for agomelatine to 3.59 (95% CI: 2.29 to 5.64) for quetiapine (Fig. 4c). Diazepam was the only treatment with fewer reported discontinuations due to AEs than placebo (RR: 0.96; 95% CI: 0.68 to 1.25).

A total of 16 treatments were included in the safety meta-analysis for AEs (alprazolam, fluoxetine, metaclazepam and sertraline were not included due to the lack of high-quality studies available). More AEs than placebo were reported for most treatments, with RRs ranging from 1.08 (95% CI: 0.89 to 1.30) for silexan to 1.72 (95% CI: 1.42 to 2.09) for diazepam (Fig. 4d). Agomelatine was the only treatment with fewer reported AEs than placebo (RR: 0.95; 95% CI: 0.79 to 1.15).

A total of 17 treatments were included in the safety meta-analysis for light AEs (fluoxetine, metaclazepam and sertraline were not included due to lack of data, incomplete data or study exclusion). More light AEs than placebo were reported for most treatments, with RRs ranging from 1.00 (95% CI: 0.69 to 1.46) for agomelatine to 3.09 (95% CI: 2.28 to 4.18) for buspirone (Fig. 4e). Clobazam was the only treatment with fewer reported light AEs than placebo (RR: 0.54; 95% CI: 0.12 to 2.48); however, the confidence interval is extremely high.

A total of 17 treatments were included in the safety meta-analysis for moderate AEs (clomipramine, metaclazepam and sertraline were not included). More moderate AEs than placebo were reported for most treatments, with RRs ranging from 1.14 (95% CI: 0.61 to 2.14) for buspirone to 6.04 (95% CI: 3.46 to 10.55) for lorazepam (Fig. 4f). Silexan was the only treatment with fewer reported moderate AEs than placebo (RR: 0.48; 95% CI: 0.22 to 1.04), albeit not statistically significant.

#### Determining the ideal therapy based on both efficacy and safety

Figure 5 illustrates the different trade-offs between efficacy and safety by plotting the network estimates for the HAM-A and total AE endpoints and moderate AEs by treatment. Notably, a treatment in the greyed region of the X-axis but outside of it on the Y-axis is most desirable. In terms of efficacy, of the most efficacious thirteen treatments, bromazepam, agomelatine, silexan, escitalopram and venlafaxine can be considered optimal treatments in terms of both efficacy and safety benefit (Fig. 5a). However, when considering the efficacy versus moderate AE trade-off, silexan was the only treatment of the best thirteen treatments in terms of efficacy that could be considered as having optimal benefit (Fig. 5b). Notably, regarding agomelatine, while the effect size is indeed comparable to silexan, the predictive and 95% confidence interval is less conclusive and more ambiguous with agomelatine than for silexan. Furthermore, while the increase of total AEs compared to placebo is low, most of this increase is concentrated in AEs of moderate severity, which is not the case for silexan.

#### Network-level diagnostics

Studies pertaining to efficacy outcomes based on HAM-A, acceptability outcomes based on all-cause discontinuations, and safety outcomes based on AEs and light AEs were identified to have a moderate degree of between-study heterogeneity (I^2^ between 30% and 60%; supplementary material_Table S7) [33]. In contrast, studies pertaining to safety outcomes based on AE-caused discontinuations and moderate AEs were identified to have a low degree of between-study heterogeneity, which may be insignificant (I^2^ between 0% and 40%; supplementary material_Table S7) [33].

#### Publication bias

The funnel plots of included trials were nearly symmetrical, suggesting no apparent small study bias or publication time bias (supplementary material_Figures S2a-i and S3a-i).

**Table 1:** Characteristics of included studies by treatment class and network

Treatment	HAM-A	Dis (all-cause)	AE-cause dis	AEs	AE of light severity	AE of moderate severity	Number of studies (*n*)	Number of randomized pts	Mean study duration (wks)	% studies with fixed-dose	% studies with flexible-dose
Placebo	X	X	X	X	X	X	77	8,311	10.2	37.7	62.3
SSRIs											
Escitalopram	X	X	X	X	X	X	17	2,429	13.2	52.9	47.1
Paroxetine	X	X	X	X	X	X	17	2,505	13.1	23.5	76.5
Sertraline	X	X	X				5	618	10.0	0.0	100.0
Fluoxetine		X	X			X	2	119	12.0	0.0	100.0
Vortioxetine	X	X	X	X	X	X	1	156	8.0	100.0	0.0
SNRIs											
Duloxetine	X	X	X	X	X	X	8	1,138	10.4	25.0	75.0
Venlafaxine	X	X	X	X	X	X	31	3,747	14.9	45.2	54.8
Anticonvulsants											
Pregabalin	X	X	X	X	X	X	18	1,550	6.7	77.8	22.2
Tricyclics											
Clomipramine	X	X	X	X	X		2	524	10.0	0.0	100.0
Benzodiazepines											
Alprazolam	X	X	X		X	X	10	449	4.8	30.0	70.0
Bromazepam	X	X	X	X	X	X	4	439	3.0	50.0	50.0
Clobazam	X	X	X	X	X	X	5	162	3.8	20.0	80.0
Diazepam	X	X	X	X	X	X	15	675	4.7	33.3	66.7
Lorazepam	X	X	X	X	X	X	9	758	5.9	44.4	55.6
Metaclazepam	X						1	25	1.9	100.0	0.0
Oxazepam	X	X	X	X	X	X	3	159	5.3	0.0	100.0
Atypical antipsychotics											
Quetiapine	X	X	X	X	X	X	3	1,583	8.6	66.7	33.3
Others											
Agomelatine	X	X	X	X	X	X	2	400	12.0	0.0	100.0
Buspirone	X	X	X	X	X	X	15	942	5.8	26.7	73.3
Silexan	X	X	X	X	X	X	6	656	9.3	100.0	0.0

*AE-cause dis* AE-caused discontinuations;* HAM-A* Hamilton Anxiety Rating Scale;* SNRIs* serotonin-norepinephrine reuptake inhibitors;* SSRIs* selective serotonin reuptake inhibitors

**Table 2:** Proportions of confidence ratings for each network treatment comparison

Network	Comparison ratings (%)				
	High	Moderate	Low	Very low	Total
HAM-A	54	34	12	0	100
AETOTAL	41	39	20	0	100
AELIG	18	58	23	1	100
AEMOD	8	20	54	18	100
DISCALL	21	65	14	1	100
DISCAE	22	61	17	0	100
Grand Total	26	47	24	3	100

*AE* adverse event;* AETOTAL* total AEs;* AELIG* light AEs;* AEMOD* moderate AEs;* DISCALL* all-cause discontinuations;* DISAE* AE-caused discontinuations;* HAM-A* Hamilton Anxiety Rating Scale

## Discussion

We performed a systematic review and NMA to compare the efficacy and acceptability evidence of anxiolytic drugs, including silexan, for the first time and to the best of our knowledge, in the treatment of anxiety disorders, including GAD. Our findings represent the most comprehensive available evidence base to guide the initial choice of treatment for anxiety disorders in adults.

Results from studies in the literature suggest that silexan may be more effective than placebo, paroxetine and lorazepam in patients with anxiety disorders, causing a greater decline in HAM-A scores across these comparators [23]. Our research methodology was similar to the systematic review and analysis conducted by Slee et al. (2019), which assessed the effectiveness of various drug treatments for adult outpatients diagnosed with GAD [24]. However, it’s worth noting that the aforementioned NMA only included adults diagnosed with GAD and did not consider any studies involving silexan [24]. We believe our study is more comprehensive and contributes to the existing body of evidence more broadly because we included adult patients in the field of GAD. Notably, we reviewed a greater number of studies than reported in Slee et al. (2019), including six studies where a total of 656 randomized patients were assigned to receive silexan versus a placebo, lorazepam, or paroxetine. This represents the first comprehensive analysis of the efficacy, acceptability, and safety of silexan, a lavender essential oil formulation, compared to other treatments for anxiety disorders.

Overall, in our systematic review and NMA, results for the 20 included pharmacological treatments were aligned with our expectations based on clinical experience. For the tricyclic clomipramine, despite having the highest efficacy, acceptability was extremely low, resulting in the highest reported all-cause discontinuations of all the treatments analyzed. For light AEs, although clobazam treatment is favored, the confidence interval limits are very wide, suggesting the results are subject to a high degree of variability or a low degree of precision. The top thirteen treatments demonstrating the best efficacy in patients with anxiety disorders were distributed across all seven medication categories: SSRIs (*n* = 1), SNRIs (*n* = 2), anticonvulsants (*n* = 1), tricyclics (*n* = 1), benzodiazepines (*n* = 5), atypical antipsychotics (*n* = 2) and phytotherapy (*n* = 1). Of these efficacious treatments, eight (diazepam, alprazolam, pregabalin, agomelatine, venlafaxine, escitalopram, silexan and bromazepam) were identified as acceptable in regard to all-cause discontinuations. Notably, the only phytomedicine (silexan) worked well across all outcomes measured. Regarding the primary outcomes analyzed (efficacy and acceptability), silexan was the sixth most efficacious treatment out of the 19 analyzed treatments and at least as acceptable as placebo in all-cause discontinuations. This indicates a good efficacy/acceptability for silexan. Furthermore, when comparing trade-offs between efficacy and safety for different treatments, among the top thirteen most effective treatments, bromazepam, agomelatine, silexan, escitalopram, and venlafaxine stand out as optimal choices considering both efficacy and safety benefits. However, when weighing efficacy against moderate AEs, silexan is the singular treatment among the top thirteen efficacious treatments that could be viewed as having an optimal benefit.

Publication bias was assessed in this review at the network level using funnel plots with standard error (scale reversed) against effect estimate (mean difference or relative risk centered at the comparison-specific estimate) with 95% confidence intervals outlining the inverted cone. We explored sources of bias along three dimensions: time bias, small-study effect and missing evidence. Visual inspection of the funnel plots did not support the presence of bias along those three dimensions, and Egger tests were usually non-significant. Egger tests, when significant, displayed negligible intercept values, which suggests to us that the large number of studies included in all networks provided power to detect lower than relevant deviation to the null and did not support evidence of bias. Study quality is also an important factor for evaluating the presence of publication bias, and it was assessed at the study level using the Rob2 framework. At the network level and as documented in our CINeMA assessment, this did decrease our confidence in some estimates. However, sensitivity analyses showed that although some comparison estimates rely heavily on individual studies, this did not overly affect the overall efficacy and acceptability estimates from the network.

While the Liebowitz scale (LSAS) is useful in certain contexts, it was not deemed the ideal choice for analyzing anxiety in this NMA because it was originally developed to assess social anxiety in older adults, and HAM-A is more standardized than LSAS [34].

Our study amalgamates existing evidence, enhancing the capacity to evaluate the effectiveness and acceptability of treatments for anxiety disorders. A key strength of our study is the consistent use of efficacy and acceptability measures (changes in HAM-A and discontinuation of the study), as variations in these outcomes can potentially lead to incorrect conclusions. Limitations should be considered when interpreting the results of this study. First, the heterogeneity between studies is substantial due to different patient populations, including age, gender, ethnic background, disease severity, inclusion or exclusion of comorbidities such as depression, etc. In addition, different study durations and flexible doses further add to the inter-study heterogeneity. Data collection of the specific AE did not include all AE reported because they were not consistently reported. We focused on 13 common terminology criteria for AEs (CTCAE). Still, sometimes only the top five AEs were reported in studies, or only those exceeding 5% or 10%, so severity CTCAEs are an undercount of the “true” severity rates. This could favor effect sizes of older drugs on all AE endpoints as reporting of AE in later studies is more homogeneous and standardized. Aspects of the studies were not pre-specified as some endpoints had to be discarded due to the non-availability of the data or the AE severity endpoint that was defined during the data collection phase. Moreover, for assessing the risk of bias (RoB2), discontinuations due to AEs were not treated as missing as the AE is known before missingness. This does not capture potential other AEs that might have occurred by continuing the study and is therefore acknowledged as a limitation. Furthermore, as defined in the literature, using the last observation carried forward (LOCF) to deal with missingness is well-known as being subpar. Still, it was universally used in all studies included. However, Baldwin et al. (2006) argue that in this use case, the estimate is actually conservative and is an approach favored by regulatory bodies in disorders such as GAD that do not deteriorate progressively [35]. 

Withdrawal symptoms during tapering, as opposed to complete discontinuation, were not analyzed in this study but remain clinically significant [36]. These symptoms can vary based on medication and patient factors, emphasizing the need for individualized tapering protocols. Future research should address this gap to better guide clinical management [36]. 

Our systematic review and NMA provides a comprehensive comparison of the efficacy and acceptability of anxiolytic drugs, including silexan, in treating anxiety disorders such as GAD. This study significantly expands the current evidence base, guiding the initial selection of treatment for adult anxiety disorders. It is worth noting that silexan, the only phytomedicine, performed well across all outcomes measured and could be viewed as having an optimal benefit in the trade-off between efficacy and moderate adverse events. Future studies should examine the long-term impacts of these treatments and their effectiveness across different demographic groups and specific diagnoses in accordance with the International Medical Health Organization (IMHO). In addition, while this study focused on adult patients, future research could expand to include pediatric and geriatric populations. These findings will contribute to a deeper understanding of treatments for anxiety disorders, enhance the quality of life for those affected, and facilitate informed decision-making processes among patients, caregivers, and medical professionals.

## Supplementary Information

Below is the link to the electronic supplementary material.

Supplementary Material 1
