# Supplementary material for: Comparative efficacy and acceptability of anxiolytic drugs for the treatment of anxiety disorders: a systematic review and network meta-analysis
Source: Eur Arch Psychiatry Clin Neurosci. 2025 Aug 11;276(4):1879–94. doi: 10.1007/s00406-025-02082-0 (PMC13234035; doi:10.1007/s00406-025-02082-0)
Supplement: Supplementary file 1 — Supplementary Material 1 [file 406_2025_2082_MOESM1_ESM.docx]

**Supplementary material**

European Archives of Psychiatry and Clinical Neuroscience

**COMPARATIVE EFFICACY AND ACCEPTABILITY OF ANXIOLYTIC DRUGS FOR THE TREATMENT OF ANXIETY DISORDERS: A SYSTEMATIC REVIEW AND NETWORK META-ANALYSIS**

Thomas J. Müller^1,2,*^, Arnaud Künzi^3^, Ellen Heitlinger^4^, Bernd Krämer^5,6^, Roland von Känel^7^, Josef Hättenschwiler^8^, Matthias Hilpert^9^, Christian Imboden^10,2^, Edith Holsboer-Trachsler^11^, Martin Hatzinger^12^, Siegfried Kasper^13,14,15^, Borwin Bandelow^16^, Erich Seifritz^17^

^1^Private Clinic Meiringen, 3860 Meiringen, Switzerland

^2^University Hospital of Psychiatry and Psychotherapy, University of Bern, Bern, Switzerland.

^3^Department of Clinical Research, University of Bern, Switzerland.

^4^H+O communications Ltd., 8041 Zurich, Switzerland.

^5^Section for Experimental Psychopathology and Neuroimaging, Department of General Psychiatry, Heidelberg University Hospital, Heidelberg, Germany.

^6^Centre for Translational Research in Systems Neuroscience and Psychiatry, Department of Psychiatry and Psychotherapy, University Medical Center Göttingen, Göttingen, Germany.

^7^Department of Consultation-Liaison-Psychiatry and Psychosomatic Medicine, University Hospital Zurich, University of Zurich, Zurich, Switzerland.

^8^Centre for Anxiety and Depression Treatment Zurich, Zurich, Switzerland.

^9^Psychiatric Services of the Canton Aargau, Windisch, Switzerland.

^10^Private Clinic Wyss, Münchenbuchsee, Switzerland.

^11^Gesundheitszentrum St. Johann, University of Basel, Basel, Switzerland.

^12^Psychiatric Services Solothurn and University of Basel, Solothurn, Switzerland.

^13^Department of Psychiatry and Psychotherapy, Medical University of Vienna, Vienna, Austria.

^14^Comprehensive Center for Clinical Neurosciences and Mental Health, Medical University of Vienna, Vienna, Austria.

^15^Center for Brain Research, Medical University of Vienna, Vienna, Austria.

^16^Department of Psychiatry and Psychotherapy, University Medical Center Göttingen, Germany.

^17^Department of Adult Psychiatry and Psychotherapy, Psychiatric Hospital, University of Zurich, Zurich, Switzerland.

***Corresponding author:** Professor Thomas J. Müller

Professor of Psychiatry, CMO

Privatklinik Meiringen,

CH - 3860 Meiringen,

Switzerland

**Email:** [thomas.mueller2@unibe.ch](mailto:thomas.mueller2@unibe.ch)

**Tel.:** [+41 33 972 81 11](tel:+41339728111)

**Fax.:** [+41 33 972 82 20](fax:+41339728220)

**ORCID ID:** 0000-0001-9315-8138

**Table S1. Dosage classification used to select which dosage arm to include in the network-meta-analysis.**

| **Drug** | **Standard dosage in CH* [mg/day]** | **Usual daily dosing range**^, #^** | **Low dose [mg/day]** | **Medium dose [mg/day]** | **High dose [mg/day]** |
| --- | --- | --- | --- | --- | --- |
| Agomelatine | 25.0 |  | < | 25.0─50.0 | > |
| Alprazolam | 0.5–4.0 |  | >0.75 | 0.75─1.5 | >1.5 |
| Bromazepam | 1.5–3.0 |  |  | 3.0─12.0 | 18.0 |
| Buspirone |  |  | 10.0 | 20.0─30.0 | 60.0 |
| Clobazam | 10.0 |  | 0.5─15.0 | 20.0─30.0 | 80.0 |
| Clomipramine | 10.0–25.0 |  | 30.0 | 50.0─100.0 | 150.0 |
| Diazepam | 5.0–10.0 |  | 2.5─10.0 | 20.0‒40.0 | 60.0 |
| Duloxetine | 30.0–60.0 | 40.0─120.0 | 30.0 | 60.0─90.0 | 120.0─150.0 |
| Escitalopram | 10.0–20.0 | 10.0–20.0 | 5.0 | 10.0 | 20.0 |
| Fluoxetine | 20.0 | 10.0–80.0 |  | 10.0 | 20.0 |
| Lorazepam | 1.0–2.5 |  | 0.5─1.0 | 2.0–4.0 | >7.5 |
| Metaclazepam |  |  | < | 15.0 | > |
| Oxazepam | 15.0 |  | 10.0 | 30.0─60.0 | 150.0 |
| Paroxetine | 20.0 | Paxil: 10.0─50.0 | 10.0 | 40.0 | 60.0 |
| Pregabalin | 25.0–300.0 |  | 75.0 | 150.0─450.0 | 600.0 |
| Quetiapin | 25.0–300.0 |  | 12.5 | 50.0─300.0 | 600.0 |
| Sertraline |  | 50.0─200.0 | 25.0 | 50.0 | 200.0 |
| Silexan | 80.0 |  | < | 80.0 | 160.0 |
| Venlafaxine | 37.5–150.0 |  | 37.5─75.0 | 75.0─150.0 | 225.0 |
| Vortioxetine | 5.0–20.0 |  | 5.0 | 10.0 | 20.0 |

*considered websites: swissmedic.ch; compendium.ch

**Hansen RA et al. (2009). Controlling for drug dose in systematic review and meta-analysis: A case study of the effect of antidepressant Dose. Med Decis Making. (<https://www.ncbi.nlm.nih.gov/pmc/articles/PMC2657322/>), Table 1.

# The usual dosing range represents doses recommended by FDA-approved product labeling. The upper bound for duloxetine was increased to 120mg/day based on practice patterns and other clinical evidence. The lower bound for some drugs was decreased (e.g., immediate-release bupropion, fluoxetine, and paroxetine) when it was deemed that the FDA-approved range did not capture the full range of doses used in clinical practice and shown to be efficacious in clinical studies.

« < » and « > » symbols without associated numbers: When only a medium dose indication was found, i.e., values below the medium dose are low and those above high, without providing a threshold.

CH, Switzerland; XR, extended-release.

**Table S2. Study arms excluded based on selected (medium) dosage classification from Table S1.**

| **Drug** | **Study ID** | **Study** "**Arm**" **excluded due to comparison of same medication + reason** |
| --- | --- | --- |
| Silexan | 1 | "Silexan 160mg": other M Silexan arm chosen |
| Escitalopram | 10  16  20 | "Escitalopram 20mg": other M Escitalopram arm chosen "Escitalopram 5mg, 20mg ": other M Escitalopram arm chosen "Escitalopram 5mg, 20mg": other M Escitalopram arm chosen |
| Quetiapine | 15  71  102 | "Quetiapine XR 300mg": other M arm chosen more in the middle of the medium range "Quetiapine XR 50mg": other M arm chosen more in the middle of the medium range "Quetiapine XR 50mg, 300mg": other M arm chosen more in the middle of the medium range |
| Buspirone | 40 | "Buspirone BID 30mg, TID 30mg": No placebo nor non-Buspirone comparator in the study |
| Vortioxetine | 51 | "Vortioxetine 2.5mg, 5mg": other M arm chosen |
| Duloxetine | 53 | "Duloxetine 20mg": other M arm chosen |
| Pregabalin | 59  61  88  89  92  93  95 | "Pregabalin 600mg": other M arm chosen "Pregabalin 600mg": other M arm chosen "Pregabalin 300mg, 600mg": other M arm chosen "Pregabalin 600mg": other M arm chosen "Pregabalin 300mg, 600mg": other M arm chosen "Pregabalin 600mg": other M arm chosen "Pregabalin 200mg, 400mg": other M arm chosen |
| Venlafaxine* | 114  119  121  123  127  128 | "Venlafaxine ER 37.5mg, 75mg": other M arm chosen "Venlafaxine fixed dose 75mg, Flexible dose 213.5mg": No placebo nor non-Venlafaxine comparator in the study "Venlafaxine XR avg 37.5, avg 150mg": other M arm chosen in that is more in the middle of the medium range "Venlafaxine XR avg 150mg": other M arm chosen "Venlafaxine XR avg 75mg, avg 225mg": other M arm chosen "Venlafaxine XR avg 150mg": other M arm chosen |

*normal range is 75-150mg. In order not to systematically choose the lighter or heavier treatment, retained arm was alternatingly 75 then 150 when encountered. Main results were robust to whichever arm was selected.

H, High; ID, identification number; M, Medium; L, Low. ID.

**Table S3. Listing of included studies by network**

| **Publication ID** | **Publication** | **HAM-A** | **Disconti­nuation** | **AE-caused discontinuation** | **Adverse events** | **AE of light severity** | **AE of moderate severity** | **DOI** |
| --- | --- | --- | --- | --- | --- | --- | --- | --- |
| 1 | Kasper (2014) | X | X | X | X |  |  | 10.1017/S1461145714000017 |
| 2 | Kasper (2015) | X | X | X | X | X |  | 10.1016/j.euroneuro.2015.12.002 |
| 3 | Kasper (2010) | X | X | X | X | X | X | 10.1097/YIC.0b013e32833b3242 |
| 4 | Kasper (2015) | X | X | X | X | X | X | 10.1016/j.euroneuro.2015.07.024 |
| 5 | Woelk (2010) | X | X | X | X | X |  | 10.1016/j.phymed.2009.10.006 |
| 7 | Lenze (2009) |  | X | X | X |  |  | 10.1001/jama.2008.977 |
| 8 | Davidson (2004) | X |  | X |  |  |  | 10.1002/da.10146 |
| 9 | Stein (2018) | X | X | X | X | X |  | 10.1016/j.euroneuro.2018.05.006 |
| 10 | Asakura (2016) |  | X | X | X | X | X | 10.1185/03007995.2016.1146663 |
| 11 | Stein (2014) |  | X | X | X | X | X | 10.4088/JCP.13m08433 |
| 12 | Kasper (2005) |  |  |  |  | X | X | 10.1192/bjp.186.3.222 |
| 13 | Bose (2008) |  | X | X |  | X | X | 10.1002/da.20355 |
| 15 | Merideth (2012) |  | X | X |  | X | X | 10.1097/YIC.0b013e32834d9f49 |
| 16 | Baldwin (2006) |  | X | X | X | X | X | 10.1192/bjp.bp.105.012799 |
| 17 | Maity (2014) |  |  |  |  |  |  | 10.4103/0253-7613.135959 |
| 20 | Lader (2004) |  | X | X | X | X | X | 10.1002/da.20014 |
| 21 | Bystritsky (2008) |  |  |  |  |  |  |  |
| 25 | Bielski (2005) |  |  | X |  | X | X | 10.1080/10401230590932326 |
| 26 | Lader (1998) |  | X |  | X | X | X | 10.1007/s002130050731 |
| 28 | Laakmann (1998) |  | X |  | X |  |  | 10.1007/s002130050578 |
| 29 | Rickels (1982) |  | X | X | X | X | X |  |
| 30 | Ansseau (1990) |  | X | X | X |  |  | 10.1159/000119464 |
| 31 | Cohn (1989) |  | X | X | X | X |  | 10.1185/03007998909115213 |
| 32 | Jacobson (1985) |  | X |  | X |  |  | 10.1002/j.1875-9114.1985.tb03430.x |
| 34 | Mokhber (2010) | X |  |  |  |  |  | 10.1111/j.1440-1819.2009.02055.x |
| 35 | Enkelmann (1991) |  | X | X | X |  |  | 10.1007/BF02244440 |
| 37 | Strand (1990) | X | X |  | X | X | X |  |
| 38 | Feighner (1982) |  |  |  | X |  | X |  |
| 39 | Ross (1987) |  |  | X |  | X |  | 10.1177/070674378703200505 |
| 40 | Sramek (1997) |  |  |  |  |  |  | 10.1016/s0149-2918(97)80134-8 |
| 41 | Böhm (1990) |  | X |  |  |  |  | 10.1097/00004714-199006001-00008 |
| 44 | Kahn (1987) | X |  |  |  |  |  | 10.1097/00004850-198701000-00003 |
| 46 | Alaka (2014) | X | X | X |  |  |  | 10.1002/gps.4088 |
| 49 | Rynn (2008) |  | X | X | X | X | X | 10.1002/da.20271 |
| 50 | Wu (2011) | X | X | X | X | X | X |  |
| 51 | Mahableshwarkar (2014) | X | X | X | X | X | X | 10.1111/ijcp.12328 |
| 53 | Nicolini (2009) | X | X | X |  | X | X | 10.1017/S0033291708003401 |
| 54 | Hartford (2007) | X | X | X | X | X | X | 10.1097/YIC.0b013e32807fb1b2 |
| 55 | Ball (2015) |  |  |  |  |  |  | 10.1017/S1041610215000381 |
| 56a | Silverstone (2001) |  | X | X |  |  | X | 10.4088/jcp.v62n07a04 |
| 56b | Silverstone (2002) |  | X | X |  |  | X | 10.4088/jcp.v62n07a05 |
| 59 | Pande (2003) |  | X | X |  | X | X | 10.1176/appi.ajp.160.3.533 |
| 60 | Herrera-Arellano (2012) |  |  |  |  |  |  | 10.1055/s-0032-1315110 |
| 61 | Feltner (2003) | X |  | X | X | X | X | 10.1097/01.jcp.0000084032.22282.ff |
| 65 | Morton (1992) |  |  |  |  |  |  | 10.1055/s-2007-1014402 |
| 66 | Ruiz (1983) |  | X | X |  |  |  |  |
| 67 | Cordingley (1985) |  | X | X | X | X | X | 10.1185/03007998509109625 |
| 68 | Möller (2001) | X | X | X |  |  |  | 10.1097/00004714-200102000-00011 |
| 71 | Bandelow (2010) |  | X | X |  | X | X | 10.1017/S1461145709990423 |
| 72 | Lepola (2004) |  | X | X |  | X | X | 10.4088/jcp.v65n0213 |
| 73 | Ball (2005) | X |  |  |  |  |  | 10.4088/jcp.v66n0113 |
| 74 | Baldwin (1999) |  | X | X | X | X | X | 10.1192/bjp.175.2.120 |
| 75 | Allgulander (2004) |  |  | X | X | X | X | 10.1002/hup.602 |
| 76 | Liebowitz (2002) |  | X | X | X | X | X | 10.4088/jcp.v63n0113 |
| 78 | Allgulander (1999) |  |  | X |  |  |  | 10.1111/j.1600-0447.1999.tb10845.x |
| 79 | Pollack (2001) |  |  | X |  | X | X | 10.4088/jcp.v62n0508 |
| 80 | Stein (1998) |  | X | X |  | X | X | 10.1001/jama.280.8.708 |
| 83 | Kim (2006) | X | X | X |  | X | X | 10.1111/j.1440-1819.2006.01512.x |
| 84 | Stein (2002) |  | X | X |  | X |  | 10.4088/jcp.v63n0211 |
| 86 | Ravindran (1997) |  | X | X | X | X |  | 10.4088/jcp.v58n0305 |
| 87 | Kasper (2009) | X | X | X |  | X | X | 10.1097/yic.0b013e32831d7980 |
| 88 | Feltner (2011) |  | X | X | X | X | X | 10.1097/YIC.0b013e32834519bd |
| 89 | Montgomery (2006) | X | X | X |  | X | X | 10.4088/jcp.v67n0511 |
| 91 | Montgomery (2008) | X | X | X |  | X | X | 10.1192/bjp.bp.107.037788 |
| 92 | Rickels (2005) | X | X | X |  | X | X | 10.1001/archpsyc.62.9.1022 |
| 93 | Pande (2004) | X | X | X |  | X | X | 10.1097/01.jcp.0000117423.05703.e7 |
| 95 | Pohl (2005) | X | X | X |  | X | X | 10.1097/01.jcp.0000155820.74832.b0 |
| 96 | Altamura (2011) | X |  |  | X |  |  | 10.1097/YIC.0b013e3283457d73 |
| 99 | Vaishnavi (2007) |  |  |  |  | X |  | 10.1016/j.pnpbp.2007.06.027 |
| 102 | Khan (2011) | X |  |  | X | X | X | 10.1097/JCP.0b013e318224864d |
| 103 | Mezhebovsky (2013) | X |  | X | X | X | X | 10.1002/gps.3867 |
| 106 | Brawman-Mintzer (2006) | X | X | X |  |  |  | 10.4088/jcp.v67n0603 |
| 108 | Allgulander (2004) | X | X | X |  |  |  | 10.1176/appi.ajp.161.9.1642 |
| 110 | Liebowitz (2003) | X | X | X |  |  |  | 10.4088/jcp.v64n0708 |
| 113 | Liebowitz (2005) |  | X | X | X | X | X | 10.4088/jcp.v66n0213 |
| 114 | Boyer (2004) |  |  |  |  |  |  | 10.1016/j.eurpsy.2004.05.010 |
| 115 | Nimatoudis (2004) | X |  |  |  | X |  | 10.1097/00004850-200411000-00003 |
| 116 | Rickels (2004) |  |  | X | X | X | X | 10.1097/01.jcp.0000138764.31106.60 |
| 117a | Montgomery (2002) | X | X | X |  |  |  | 10.1097/00004714-200212000-00005 |
| 118 | Lenox-Smith (2003) | X | X |  | X | X | X |  |
| 119 | Stein (2005) |  |  |  |  | X | X | 10.1007/s00213-004-1957-9 |
| 121 | Allgulander (2001) |  | X | X |  | X |  | 10.1192/bjp.179.1.15 |
| 122 | Gelenberg (2000) | X | X | X |  | X | X | 10.1001/jama.283.23.3082 |
| 123 | Hackett (2003) |  | X | X |  |  |  | 10.1016/s0924-9338(03)00046-4 |
| 127 | Rickels (2000) |  | X | X |  | X | X | 10.1176/appi.ajp.157.6.968 |
| 128 | Davidson (1999) | X |  |  |  | X |  | 10.4088/jcp.v60n0805 |
| 131 | Väisänen (1987) | X | X |  |  | X |  | 10.1111/j.1600-0447.1987.tb02830.x |
| 132 | Elie (1984) | X |  |  |  |  |  |  |
| 134 | Aden (1980) |  | X | X |  |  |  |  |
| 135 | Chouinard (1982) |  | X |  |  | X |  | 10.1007/BF00464571 |
| 136 | Dunner (1986) | X | X | X |  |  |  |  |
| 137 | Kerry (1983) | X | X |  |  |  |  |  |
| 138 | Fontaine (1986) | X | X |  |  | X |  | 10.1111/j.1600-0447.1986.tb06268.x |
| 139 | Fontaine (1983) |  | X |  |  | X | X |  |
| 141 | Bilone (1988) | X |  |  |  |  |  | 10.1185/03007998809111130 |
| 145 | Rickels (1993) |  |  |  |  | X |  | 10.1001/archpsyc.1993.01820230054005 |
| 146 | Lapierre (1982) | X | X |  | X |  |  |  |
| 147 | Botter (1980) |  |  |  |  |  |  | 10.1185/03007998009109494 |
| 148 | Rickels (1981) |  | X | X |  |  |  | 10.1038/clpt.1981.132 |
| 149 | Jacobson (1983) |  | X | X | X | X | X |  |

**Table S4. Listing of excluded studies**

| **Publication ID** | **Publication** | **Reason** | **DOI** |
| --- | --- | --- | --- |
| 6 | Kasper (2017) | pooled dataset of publ 001 and 004 | 10.1097/YIC.0000000000000176 |
| 14 | Montgomery (2005) | includes a 12 week of open-label treatment phase, also includes SDS score however it is split in work, social and family life | 10.4088/jcp.v66n1009 |
| 18 | Goodman (2005) | data is pooled from three separate studies | 10.1016/j.jad.2004.11.011 |
| 19 | Stein (2005) | Same study as 18 "The effects of escitalopram on the HAMA have been previously described in detail" | 10.1080/10401230590932335 |
| 22 | Demyttenaere (2008) | Review paper "data from eight randomized, 8-week, double-blind, placebo-controlled clinical trials with escitalopram were used." | 10.1097/YIC.0b013e328303ac5f |
| 23 | Allgulander (2007) | SF-36 mental and physical health data combined "this tool measures eight subscales. Four are mental health related and four are physical health related" | 10.1185/030079907X226087 |
| 24 | François (2008) | SF-36 mental and physical health data combined "this is a generic instrument providing eight domain scores" | 10.1111/j.1742-1241.2008.01879.x |
| 27 | Petracca (1990) |  |  |
| 33 | Olajide (1987) | exclude: all patients received all 3 drugs successively (buspirone, diazepam and placebo) |  |
| 36 | Lemoine (1996) | exclude: 2 phases and wash-out during study in 2/4 groups |  |
| 42 | Lenze (2005) | 12/34 patients took 2mg of lorazepam for the period of the study, because they were already on this medication | 10.1176/appi.ajp.162.1.146 |
| 43 | Blank (2006) | excluded | 10.4088/jcp.v67n0319 |
| 45 | Lemoine (1991) |  | 10.1055/s-2007-1014464 |
| 47 | Davidson (2008) | Study with open-label Phase I with Duloxetine; baseline; randomized double-blind Phase II with Duloxetine and placebo | 10.1016/j.euroneuro.2008.05.002 |
| 48 | Simon (2010) | Study with open-label Phase I treated with Duloxetine; baseline; randomized double-blind Phase II trated with Duloxetine and placebo |  |
| 52 | Endicott (2007) | Study composed of three different studies | 10.4088/jcp.v68n0405 |
| 57 | Londborg (2000) |  | 10.1016/s0165-0327(99)00195-0 |
| 58 | Tiller (1997) | not included -- 2 incomplete studies in 1 publication, not usable |  |
| 62 | Kasper (2014) |  | 10.1017/S1461145713001557 |
| 63 | DelleChiaie (1995) |  | 10.1097/00004714-199502000-00003 |
| 64 | Spénard (1988) | not included because of unclear data, only 12 participants | 10.1002/bod.2510090504 |
| 69 | Parris (2018) | no end point data available for any of the measures we were looking for | 10.1097/YIC.0000000000000225 |
| 70 | Liebowitz (2005) |  | 10.1001/archpsyc.62.2.190 |
| 77 | Stein (1999) |  |  |
| 81 | Stocchi (2003) | no CGI data; see Study_info_rob2 sheet for exclusion rationale | 10.4088/jcp.v64n0305 |
| 82 | Stein (2002) | data pooled from 3 different studies. The article itself lacked data in multiple categories and only had data on cgi scores | 10.1001/archpsyc.59.12.1111 |
| 85 | Stein (2006) | Pooled data. Main drug is escitalopram. | 10.1016/j.euroneuro.2005.05.004 |
| 90 | Feltner (2008) | Has an open label phase which heavily skews results | 10.1097/YIC.0b013e3282f0f0d7 |
| 94 | Griest (2011) | Has an open label phase which heavily skews results | 10.1097/YIC.0b013e3283491fd5 |
| 97 | Katzmann (2011) | Has an open label phase in which the HAMA scores drops heavily | 10.1097/YIC.0b013e32833e34d9 |
| 98 | Sheehan (2013) |  | 10.1016/j.jad.2013.07.037 |
| 100 | Khan (2014) |  |  |
| 101 | Khan (2013) |  |  |
| 104 | Montgomery (2014) | exclude: pooled, post-hoc analysis of 3 studies | 10.1097/YIC.0000000000000026 |
| 105 | Stein (2011) | exclude: pooled, post-hoc analysis of 3 studies | 10.1002/hup.1256 |
| 107 | Dahl (2005) | exclude -- same patient population as 108, less comprehensive | 10.1111/j.1600-0447.2005.00529.x |
| 109 | Pollack (2014) | only LSAS | 10.1176/appi.ajp.2013.12101353 |
| 111 | Steiner (2005) | exclude -- same patient population as 108 | 10.1002/hup.648 |
| 112 | Connor (2006) | exclude: Combination of data from 2 trials | 10.1002/da.20086 |
| 117b | Montgomery (2002) | ak: no info available | 10.1097/00004714-200212000-00005 |
| 120 | Rickels (2010) | Has 2 phases for double blind trail, would need another column to look enter data for second and third phase | 10.1001/archgenpsychiatry.2010.170 |
| 124 | Baldwin (2012) | data pooled from 4 different studies | 10.1016/j.euroneuro.2011.07.005 |
| 125 | Meoni (2004) | 5 studies, 2 with long term extensions | 10.1002/da.10141 |
| 126 | Lydiard (1999) | not able to retreive PDF | 10.3810/pgm.11.1999.suppl1.3 |
| 129 | Feighner (1999) | not able to retreive PDF | 10.3810/pgm.11.1999.suppl1.2 |
| 130 | Hadley (2012) | exclude: premedication, taper-off study. In this study other drugs are involved in the two arms | 10.1177/0269881111405360 |
| 133 | Freeman (1986) | exclude: This study doesn not take into account any of the interested outcomes |  |
| 140 | Schuck (1998) | it doesn not analyse any of the outcomes | 10.1111/j.1472-8206.1998.tb00973.x |
| 142 | Fontaine (1984) | excluded: no usable values reportet. + might be the same study population as 139 |  |
| 143 | Dorman (1983) | excluded: withdrawal study |  |
| 144 | Jacobson (1985) | Same publication as Buspirone 32 |  |
| 150 | Bollu (2010) | excluded: same study population as publication #087 | 10.4321/S0213-61632010000100003 |

**Table S5. Study characteristics, including direct treatment comparisons**

|  | | | **N** | | **Study length** | | **Direct comparisons available** | | | | | |
| --- | --- | --- | --- | --- | --- | --- | --- | --- | --- | --- | --- | --- |
|  | | | **Studies** | **Patients randomized** | **Average** | **Weighted average** | **HAM-A** | **Discontinuation** | **AE Discontinuation** | **AE** | **AE - light** | **AE - moderate** |
| SSRIs | Escitalopram | Placebo | 9 | 2,826 | 12.0 | 12.0 | X | X | X | X | X | X |
|  | Paroxetine | Placebo | 13 | 3,771 | 13.2 | 13.3 | X | X | X | X | X | X |
|  |  | Escitalopram | 3 | 734 | 20.0 | 19.5 |  | X | X | X | X | X |
|  |  | Quetiapine | 1 | 435 | 10.0 | 10.0 |  | X | X |  | X | X |
|  |  | Venlafaxine | 1 | 269 | 12.0 | 12.0 | X | X | X | X | X | X |
|  | Sertraline | Placebo | 3 | 1,131 | 11.3 | 11.4 | X | X | X |  |  |  |
|  |  | Buspirone | 1 | 46 | 8.0 | 8.0 | X |  |  |  |  |  |
|  |  | Paroxetine | 1 | 53 | 8.0 | 8.0 | X |  |  |  |  |  |
|  | Fluoxetine | Placebo | 2 | 237 | 12.0 | 12.0 |  | X | X |  |  | X |
|  | Vortioxetine | Placebo | 1 | 313 | 8.0 | 8.0 | X | X | X | X | X | X |
| SNRIs | Duloxetine | Placebo | 7 | 2,083 | 10.4 | 10.2 | X | X | X | X | X | X |
|  |  | Vortioxetine | 1 | 312 | 8.0 | 8.0 | X | X | X |  |  |  |
|  | Venlafaxine | Placebo | 21 |  | 14.2 |  | X | X | X | X | X | X |
|  |  | Duloxetine | 2 | 653 | 10.0 | 10.0 | X | X | X | X | X | X |
|  |  | Escitalopram | 1 | 264 | 8.0 | 8.0 |  | X | X |  |  |  |
|  |  | Fluoxetine | 2 | 241 | 12.0 | 12.0 |  | X | X |  |  | X |
|  |  | Paroxetine | 3 | 643 | 10.7 | 11.6 | X | X | X | X | X | X |
|  |  | Pregabalin | 2 | 456 | 7.0 | 7.1 | X | X | X |  | X | X |
| Anticonvulsants | Pregabalin | Placebo | 9 | 1,607 | 6.7 | 6.7 | X | X | X | X | X | X |
| Tricyclics | Buspirone | Placebo | 8 | 970 | 5.4 | 5.3 | X | X | X | X | X | X |
|  |  | Diazepam | 1 | 118 | 4.0 | 4.0 |  | X | X | X | X | X |
|  |  | Oxazepam | 1 | 230 | 6.0 | 6.0 | X | X | X | X | X | X |
|  |  | Venlafaxine | 1 | 200 | 8.0 | 8.0 | X |  |  |  | X |  |
|  | Clomipramine | Placebo | 1 | 36 | 8.0 | 8.0 | X |  |  |  |  |  |
|  |  | Paroxetine | 1 | 1,019 | 12.0 | 12.0 |  | X | X | X | X |  |
| Benzodiazepines | Lorazepam | Placebo | 4 | 379 | 5.5 | 5.1 | X | X | X | X | X | X |
|  |  | Alprazolam | 1 | 53 | 4.0 | 4.0 |  | X | X |  |  |  |
|  |  | Bromazepam | 1 | 40 | 4.0 | 4.0 | X | X | X | X | X | X |
|  |  | Buspirone | 1 | 115 | 10.0 | 10.0 |  | X |  | X |  |  |
|  |  | Pregabalin | 2 | 275 | 4.0 | 4.0 | X | X | X |  | X | X |
|  | Alprazolam | Placebo | 7 | 721 | 5.1 | 4.5 | X | X | X |  | X | X |
|  |  | Buspirone | 1 | 63 | 6.0 | 6.0 |  | X | X |  |  |  |
|  |  | Oxazepam | 1 | 60 | 4.0 | 4.0 | X | X |  |  | X |  |
|  |  | Pregabalin | 1 | 184 | 4.0 | 4.0 | X | X | X |  | X | X |
|  | Bromazepam | Placebo | 2 | 72 | 4.0 | 4.0 | X | X |  |  | X | X |
|  |  | Lorazepam | 1 | 750 | 2.0 | 2.0 | X | X | X | X | X | X |
|  | Diazepam | Placebo | 10 | 1,125 | 5.0 | 5.1 |  | X | X | X | X | X |
|  |  | Alprazolam | 3 | 243 | 4.7 | 4.3 | X | X | X |  |  |  |
|  |  | Bromazepam | 1 | 32 | 4.0 | 4.0 |  | X |  |  | X | X |
|  |  | Buspirone | 4 | 493 | 4.0 | 4.0 |  | X | X | X | X | X |
|  |  | Clobazam | 2 | 199 | 4.0 | 4.0 | X | X | X | X | X | X |
|  |  | Venlafaxine | 1 | 280 | 8.0 | 8.0 |  | X | X |  |  |  |
|  | Clobazam | Placebo | 3 | 231 | 3.7 | 3.8 |  | X | X | X | X | X |
|  |  | Buspirone | 1 | 40 | 3.0 | 3.0 |  |  |  |  |  |  |
|  |  | Diazepam | 2 | 85 | 4.0 | 4.0 | X | X | X | X | X | X |
|  | Metaclazepam | Bromazepam | 1 | 50 | 1.9 | 1.9 | X |  |  |  |  |  |
|  | Oxazepam | Buspirone | 1 | 26 | 6.0 | 6.0 | X | X | X | X | X | X |
| Atypical antipsychotics | Quetiapine | Placebo | 6 | 1,830 | 8.5 | 8.7 | X | X | X | X | X | X |
|  |  | Escitalopram | 1 | 432 | 8.0 | 8.0 |  | X | X |  | X | X |
|  | Agomelatine | Placebo | 1 | 270 | 12.0 | 12.0 |  | X | X | X | X | X |
|  |  | Escitalopram | 2 | 804 | 12.0 | 12.0 | X | X | X | X | X | X |
| Plant-based | Silexan | Placebo | 4 | 975 | 10.0 | 10.0 | X | X | X | X | X | X |
|  |  | Lorazepam | 1 | 77 | 6.0 | 6.0 | X | X | X | X | X |  |
|  |  | Paroxetine | 1 | 272 | 10.0 | 10.0 | X | X | X | X |  |  |
| Multiarm studies and studies without placebo appear in the tally more than once. | | | | | | | | | | | | |

AEs, adverse events; HAM-A, Hamilton Anxiety Rating Scale.

**Table S6. Study and patient characteristics, including direct treatment comparisons**

|  | | | **Unweighted (study averages/proportions)** | | | | | | **Weighted (Patient averages/proportions)** | | | | | |
| --- | --- | --- | --- | --- | --- | --- | --- | --- | --- | --- | --- | --- | --- | --- |
|  | | | **analyzed patients (%)** | **missing # analyzed patients (%)** | **mean age** | **missing age** | **proportion of females (%)** | **missing gender** | **analyzed patients (%)** | **missing # analyzed patients (%)** | **mean age** | **missing age** | **proportion of females (%)** | **missing gender** |
| SSRIs | Escitalopram | Placebo | 99.2 | 0.0 | 42.1 | 0.0 | 59.6 | 0.0 | 99.2 | 0.0 | 40.3 | 0.0 | 58.7 | 0.0 |
|  | Paroxetine | Placebo | 98.6 | 0.0 | 39.0 | 0.0 | 54.1 | 0.0 | 98.6 | 0.0 | 39.1 | 0.0 | 54.2 | 0.0 |
|  |  | Escitalopram | 100.0 | 0.0 | 38.7 | 0.0 | 60.4 | 0.0 | 100.0 | 0.0 | 38.9 | 0.0 | 59.7 | 0.0 |
|  |  | Quetiapine | 98.9 | 0.0 | 42.0 | 0.0 | 64.8 | 0.0 | 98.9 | 0.0 | 42.0 | 0.0 | 64.8 | 0.0 |
|  |  | Venlafaxine | 100.0 | 0.0 | 35.8 | 0.0 | 46.1 | 0.0 | 100.0 | 0.0 | 35.8 | 0.0 | 46.1 | 0.0 |
|  | Sertraline | Placebo | 98.1 | 0.0 | 38.9 | 0.0 | 50.4 | 0.0 | 98.2 | 0.0 | 38.8 | 0.0 | 49.9 | 0.0 |
|  |  | Buspirone | 100.0 | 0.0 | 66.5 | 0.0 | 56.5 | 0.0 | 100.0 | 0.0 | 66.5 | 0.0 | 56.5 | 0.0 |
|  |  | Paroxetine | 100.0 | 0.0 | 39.5 | 0.0 | 77.4 | 0.0 | 100.0 | 0.0 | 39.5 | 0.0 | 77.4 | 0.0 |
|  | Fluoxetine | Placebo | 100.0 | 0.0 | 42.9 | 0.0 | 62.1 | 0.0 | 100.0 | 0.0 | 42.4 | 0.0 | 58.6 | 0.0 |
|  | Vortioxetine | Placebo | 100.0 | 0.0 | 38.3 | 0.0 | 66.1 | 0.0 | 100.0 | 0.0 | 38.3 | 0.0 | 66.1 | 0.0 |
| SNRIs | Duloxetine | Placebo | 100.1 | 14.3 | 46.0 | 28.6 | 64.3 | 28.6 | 100.1 | 15.7 | 46.1 | 29.7 | 65.0 | 29.7 |
|  |  | Vortioxetine | 100.0 | 0.0 | 39.6 | 0.0 | 69.9 | 0.0 | 100.0 | 0.0 | 39.6 | 0.0 | 69.9 | 0.0 |
|  | Venlafaxine | Placebo | 97.1 | 14.3 | 41.4 | 14.3 | 54.7 | 14.3 | 96.8 |  | 41.2 |  | 52.9 |  |
|  |  | Duloxetine | 100.0 | 50.0 | 40.2 | 50.0 | 63.2 | 50.0 | 100.0 | 50.1 | 40.2 | 50.1 | 63.2 | 50.1 |
|  |  | Escitalopram | 97.0 | 0.0 | 37.6 | 0.0 | 60.2 | 0.0 | 97.0 | 0.0 | 37.6 | 0.0 | 60.2 | 0.0 |
|  |  | Fluoxetine | 100.0 | 0.0 | 42.5 | 0.0 | 62.7 | 0.0 | 100.0 | 0.0 | 42.1 | 0.0 | 61.8 | 0.0 |
|  |  | Paroxetine | 85.7 | 0.0 | 39.6 | 0.0 | 44.0 | 0.0 | 89.0 | 0.0 | 37.9 | 0.0 | 43.1 | 0.0 |
|  |  | Pregabalin | 100.0 | 0.0 | 43.3 | 0.0 | 55.9 | 0.0 | 100.0 | 0.0 | 43.1 | 0.0 | 55.4 | 0.0 |
| Anticonvulsants | Pregabalin | Placebo | 99.9 | 0.0 | 43.0 | 11.1 | 54.8 | 11.1 | 99.9 | 0.0 | 45.4 | 10.8 | 57.3 | 10.8 |
| Tricyclics | Buspirone | Placebo | 97.8 | 0.0 | 39.7 | 12.5 | 64.6 | 25.0 | 96.9 | 0.0 | 39.4 | 6.4 | 69.6 | 8.6 |
|  |  | Diazepam | 84.7 | 0.0 | 38.7 | 0.0 | 65.3 | 0.0 | 84.7 | 0.0 | 38.7 | 0.0 | 65.3 | 0.0 |
|  |  | Oxazepam | 100.0 | 0.0 | 45.0 | 0.0 | 78.3 | 0.0 | 100.0 | 0.0 | 45.0 | 0.0 | 78.3 | 0.0 |
|  |  | Venlafaxine | 90.0 | 0.0 | 37.5 | 0.0 | 51.5 | 0.0 | 90.0 | 0.0 | 37.5 | 0.0 | 51.5 | 0.0 |
|  | Clomipramine | Placebo | 83.3 | 0.0 |  | 100.0 |  | 100.0 | 83.3 | 0.0 |  | 100.0 |  | 100.0 |
|  |  | Paroxetine | 98.3 | 0.0 | 42.7 | 0.0 | 72.2 | 0.0 | 98.3 | 0.0 | 42.7 | 0.0 | 72.2 | 0.0 |
| Benzodiazepines | Lorazepam | Placebo | 100.0 | 0.0 | 41.0 | 25.0 | 63.6 | 25.0 | 100.0 | 0.0 | 39.2 | 10.6 | 62.2 | 10.6 |
|  |  | Alprazolam | 100.0 | 0.0 | 36.9 | 0.0 | 67.9 | 0.0 | 100.0 | 0.0 | 36.9 | 0.0 | 67.9 | 0.0 |
|  |  | Bromazepam | 100.0 | 0.0 |  | 100.0 |  | 100.0 | 100.0 | 0.0 |  | 100.0 |  | 100.0 |
|  |  | Buspirone | 100.0 | 0.0 | 50.0 | 0.0 | 63.5 | 0.0 | 100.0 | 0.0 | 50.0 | 0.0 | 63.5 | 0.0 |
|  |  | Pregabalin | 100.0 | 0.0 | 37.2 | 0.0 | 55.6 | 0.0 | 100.0 | 0.0 | 37.2 | 0.0 | 55.6 | 0.0 |
|  | Alprazolam | Placebo | 100.0 | 28.6 | 37.0 | 42.9 | 68.9 | 42.9 | 100.0 | 50.2 | 38.8 | 58.9 | 66.6 | 58.9 |
|  |  | Buspirone | 100.0 | 0.0 |  | 100.0 |  | 100.0 | 100.0 | 0.0 |  | 100.0 |  | 100.0 |
|  |  | Oxazepam | 100.0 | 0.0 | 35.2 | 0.0 | 56.7 | 0.0 | 100.0 | 0.0 | 35.2 | 0.0 | 56.7 | 0.0 |
|  |  | Pregabalin | 99.5 | 0.0 | 39.0 | 0.0 | 62.0 | 0.0 | 99.5 | 0.0 | 39.0 | 0.0 | 62.0 | 0.0 |
|  | Bromazepam | Placebo | 100.0 | 50.0 |  | 100.0 |  | 100.0 | 100.0 | 44.4 |  | 100.0 |  | 100.0 |
|  |  | Lorazepam | 100.0 | 0.0 |  | 100.0 | 72.4 | 0.0 | 100.0 | 0.0 |  | 100.0 | 72.4 | 0.0 |
|  | Diazepam | Placebo | 96.5 | 30.0 | 37.5 | 40.0 | 61.1 | 50.0 | 96.6 | 26.2 | 39.0 | 36.1 | 66.7 | 37.9 |
|  |  | Alprazolam | 100.0 | 66.7 | 31.5 | 66.7 | 68.8 | 66.7 | 100.0 | 86.8 | 31.5 | 86.8 | 68.8 | 86.8 |
|  |  | Bromazepam |  | 100.0 |  | 100.0 |  | 100.0 |  | 100.0 |  | 100.0 |  | 100.0 |
|  |  | Buspirone | 92.7 | 0.0 | 37.9 | 25.0 | 71.0 | 50.0 | 93.3 | 0.0 | 38.1 | 13.4 | 77.0 | 17.2 |
|  |  | Clobazam | 86.7 | 50.0 | 38.4 | 50.0 | 40.0 | 50.0 | 86.7 | 54.8 | 38.4 | 54.8 | 40.0 | 54.8 |
|  |  | Venlafaxine | 100.0 | 0.0 | 44.7 | 0.0 | 66.0 | 0.0 | 100.0 | 0.0 | 44.7 | 0.0 | 66.0 | 0.0 |
|  | Clobazam | Placebo | 92.6 | 33.3 | 36.8 | 33.3 | 52.3 | 33.3 | 89.8 | 44.6 | 37.0 | 44.6 | 48.4 | 44.6 |
|  |  | Buspirone | 100.0 | 0.0 | 37.3 | 0.0 | 60.0 | 0.0 | 100.0 | 0.0 | 37.3 | 0.0 | 60.0 | 0.0 |
|  |  | Diazepam | 100.0 | 0.0 | 38.2 | 0.0 | 58.9 | 0.0 | 100.0 | 0.0 | 38.4 | 0.0 | 58.8 | 0.0 |
|  | Metaclazepam | Bromazepam | 100.0 | 0.0 |  | 100.0 |  | 100.0 | 100.0 | 0.0 |  | 100.0 |  | 100.0 |
|  | Oxazepam | Buspirone | 100.0 | 0.0 | 40.2 | 0.0 | 53.8 | 0.0 | 100.0 | 0.0 | 40.2 | 0.0 | 53.8 | 0.0 |
| Atypical antipsychotics | Quetiapine | Placebo | 98.5 | 0.0 | 48.5 | 16.7 | 66.6 | 16.7 | 97.8 | 0.0 | 47.5 | 0.8 | 64.6 | 0.8 |
|  |  | Escitalopram | 96.1 | 0.0 | 39.3 | 0.0 | 63.9 | 0.0 | 96.1 | 0.0 | 39.3 | 0.0 | 63.9 | 0.0 |
|  | Agomelatine | Placebo | 100.0 | 0.0 | 43.3 | 0.0 | 73.3 | 0.0 | 100.0 | 0.0 | 43.3 | 0.0 | 73.3 | 0.0 |
|  |  | Escitalopram | 100.0 | 0.0 | 41.7 | 0.0 | 70.3 | 0.0 | 100.0 | 0.0 | 41.5 | 0.0 | 69.9 | 0.0 |
| Plant-based | Silexan | Placebo | 99.2 | 0.0 | 46.9 | 0.0 | 75.6 | 0.0 | 99.2 | 0.0 | 46.8 | 0.0 | 76.6 | 0.0 |
|  |  | Lorazepam |  | 100.0 |  | 100.0 |  | 100.0 |  | 100.0 |  | 100.0 |  | 100.0 |
|  |  | Paroxetine | 98.2 | 0.0 | 45.8 | 0.0 | 72.4 | 0.0 | 98.2 | 0.0 | 45.8 | 0.0 | 72.4 | 0.0 |
| Denominator: number of randomized patients | | | | | | | | | | | | | | |
| For unweighted figures, the statistical unit is the study. For weighted figures, the statistical unit is the patient. | | | | | | | | | | | | | | |

**Figure S1. Included and excluded studies based on efficacy and safety outcome measures.**


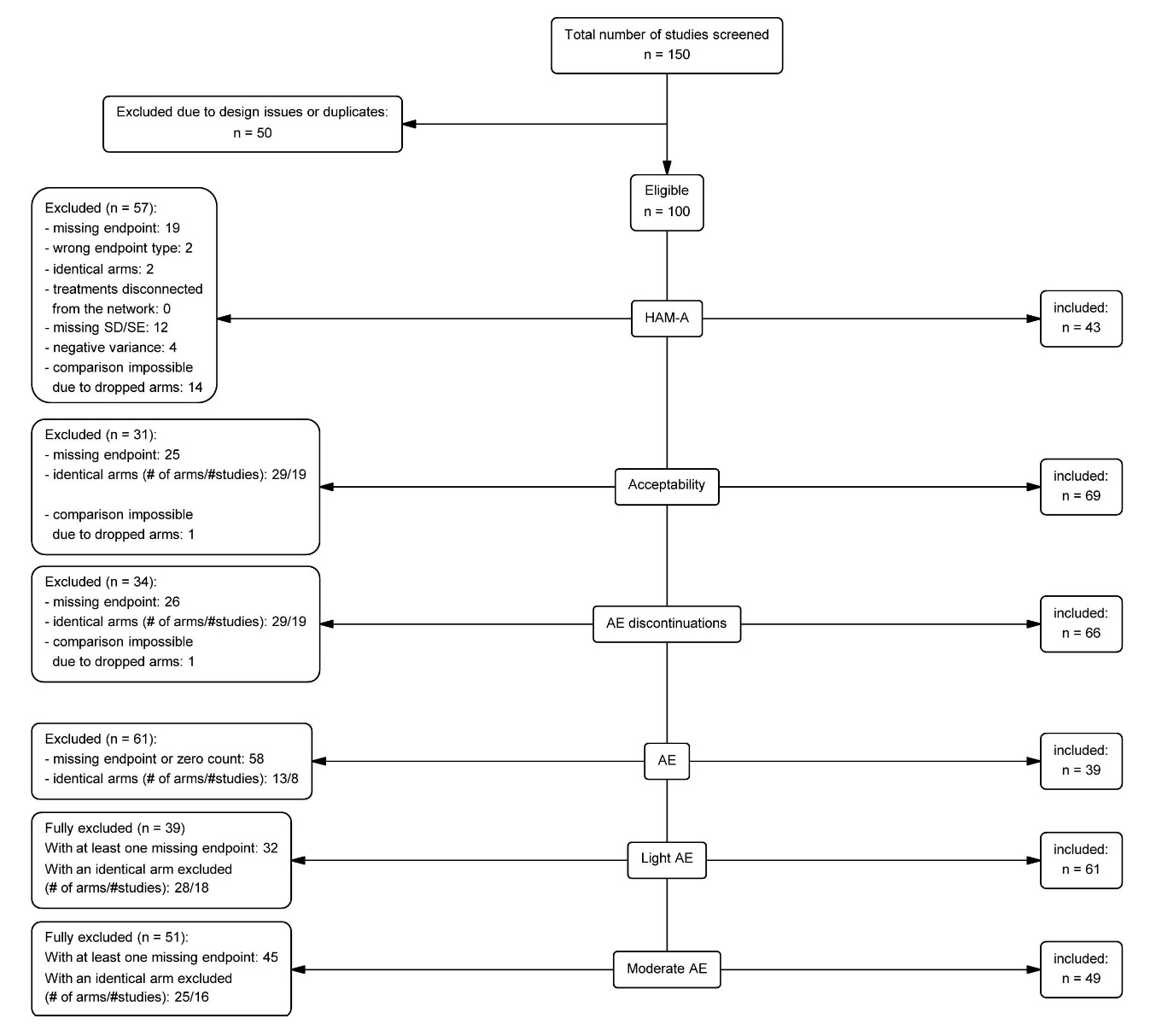


AEs, adverse events; HAM-A, Hamilton Anxiety Rating Scale.

**Table S7. Network-level diagnostics for heterogeneity**

AEs, adverse events; HAM-A, Hamilton Anxiety Rating Scale.

| **Metric** | **HAM-A** | **All-cause discontinuations** | **AE-caused discontinuations** | **AEs** | **Light AEs** | **Moderate AEs** |
| --- | --- | --- | --- | --- | --- | --- |
| **tau** | 1.33 | 0.21 | 0.259 | 0.078 | 0.208 | 0.241 |
| **tau^2^** | 1.77 | 0.044 | 0.067 | 0.006 | 0.043 | 0.058 |
| **I^2^** | 62.6% | 43.4% | 23.2% | 46% | 53.6% | 27.7% |

**Figure S2. Funnel plots – small study bias (ordered by number of randomized patients)**

| **a) HAM-A**  **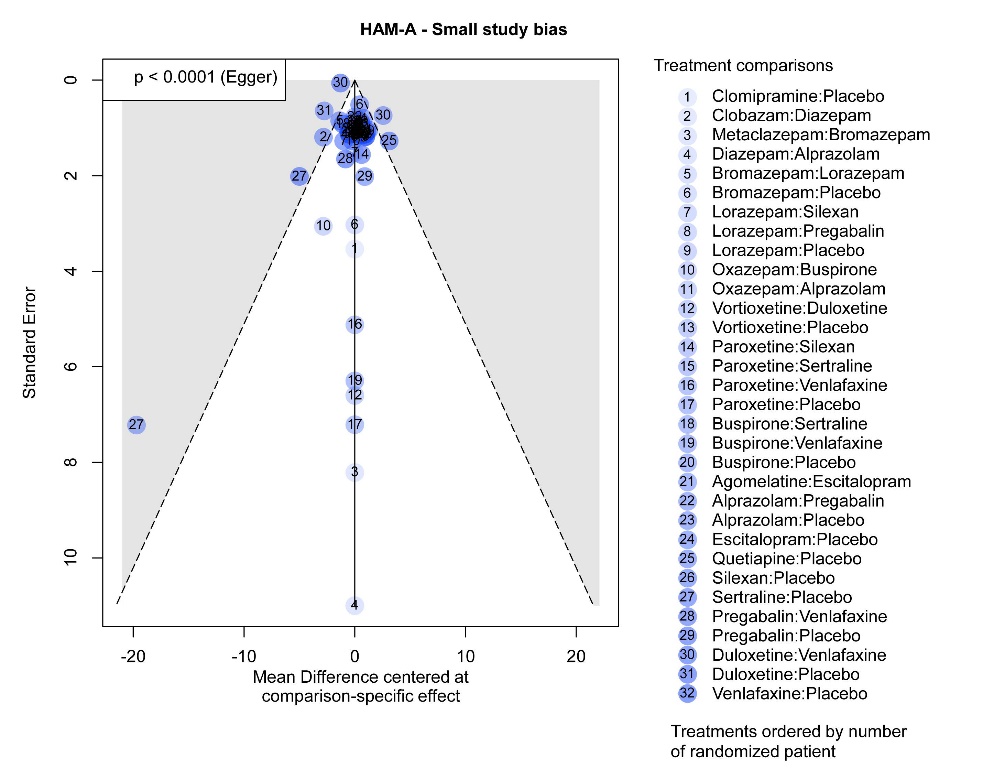** |
| --- |
| **b) HAM-A (zoomed in)**  **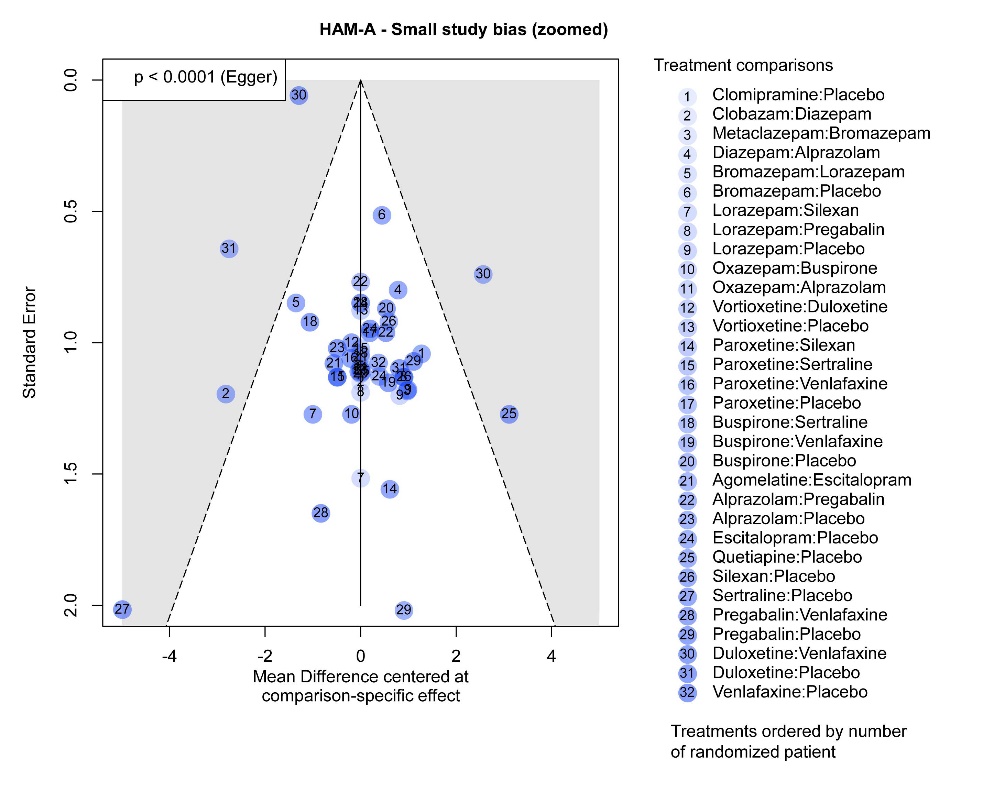** |
| **c) All-cause discontinuations**  **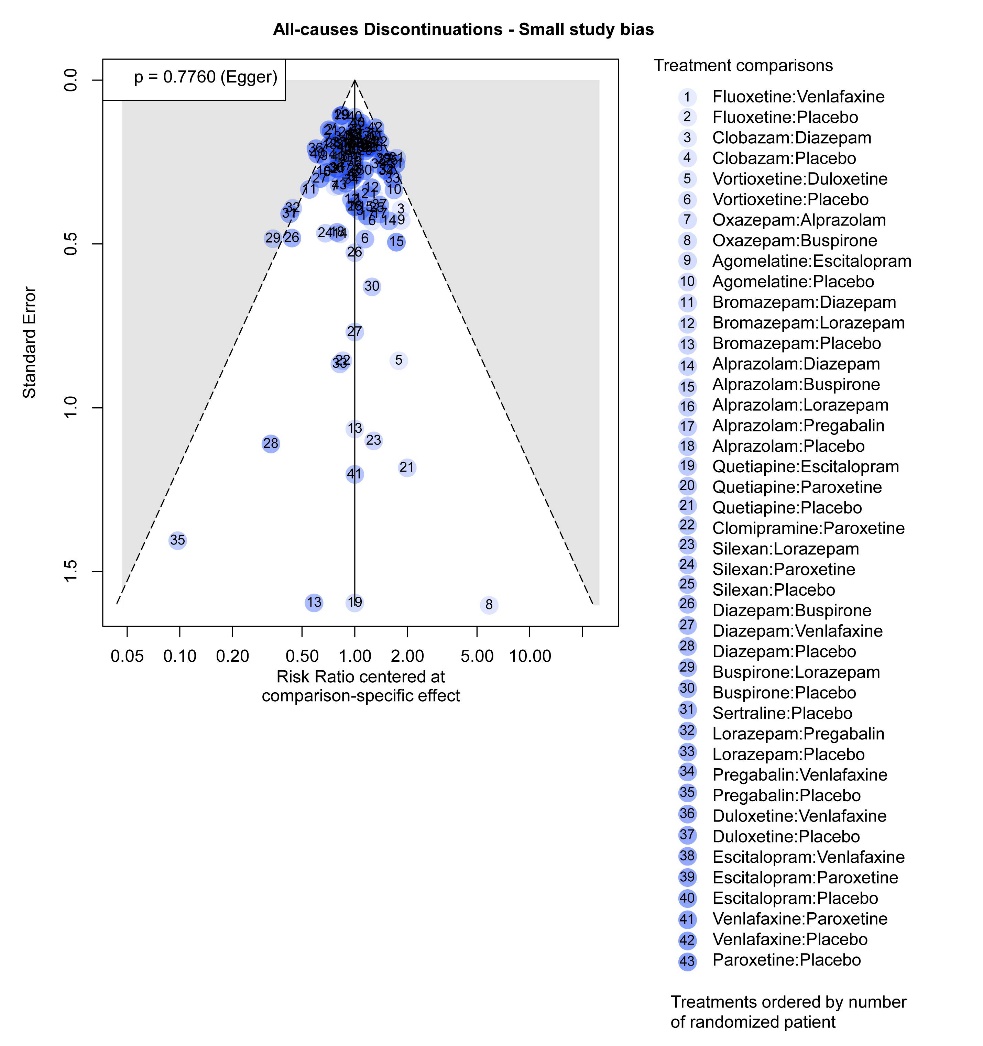** |
| **d) All-cause discontinuations (zoomed in)**  **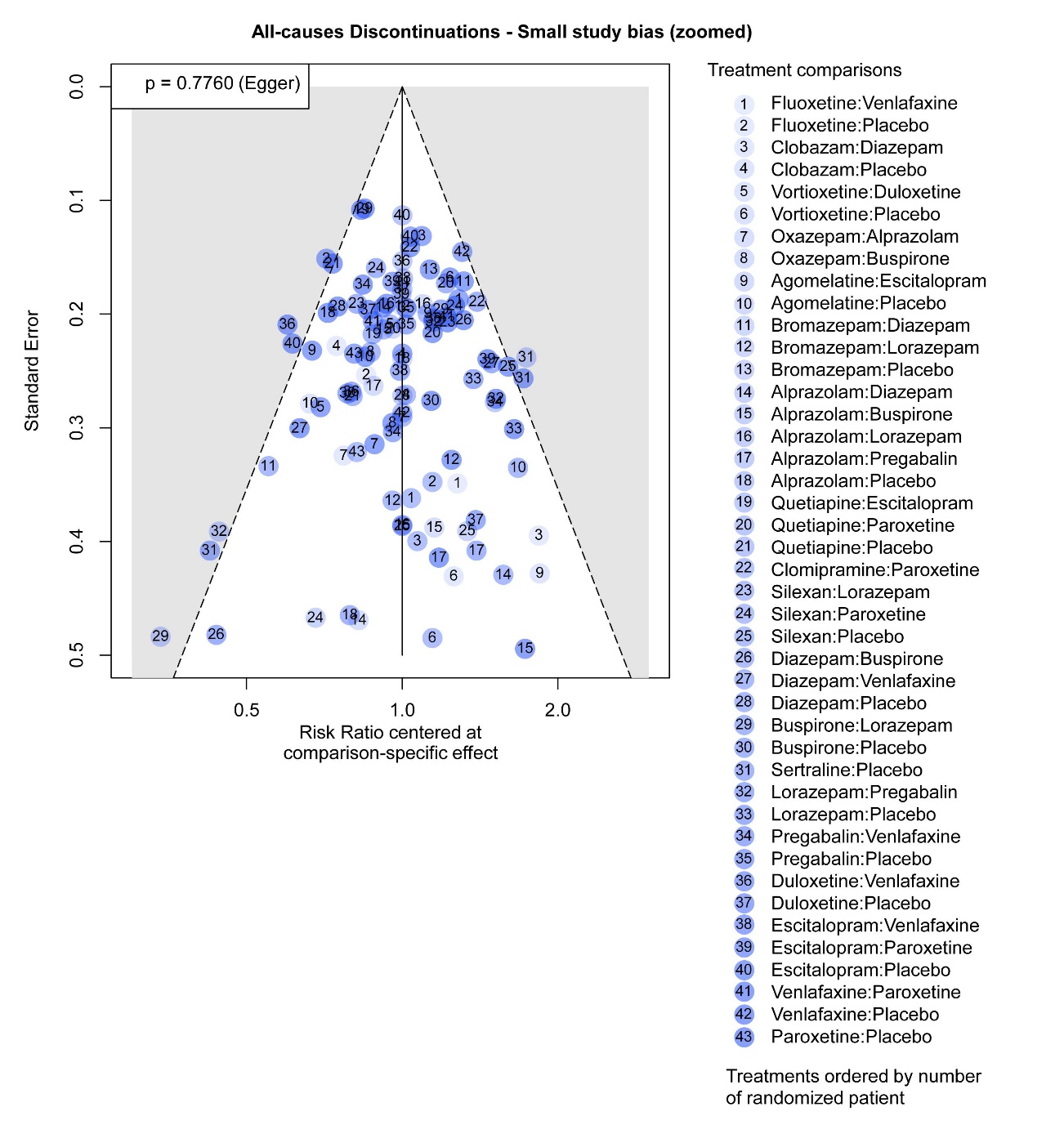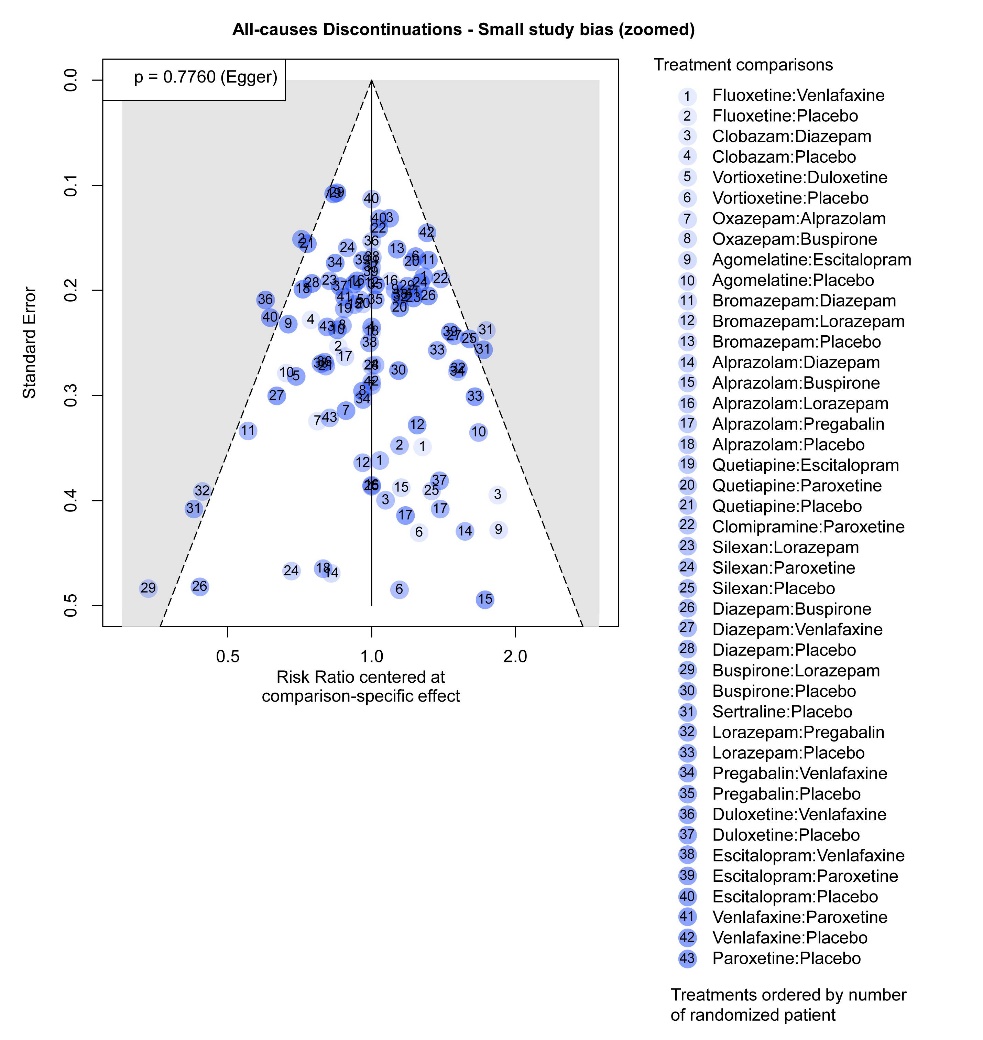** |
| **e) AE-caused discontinuations**  **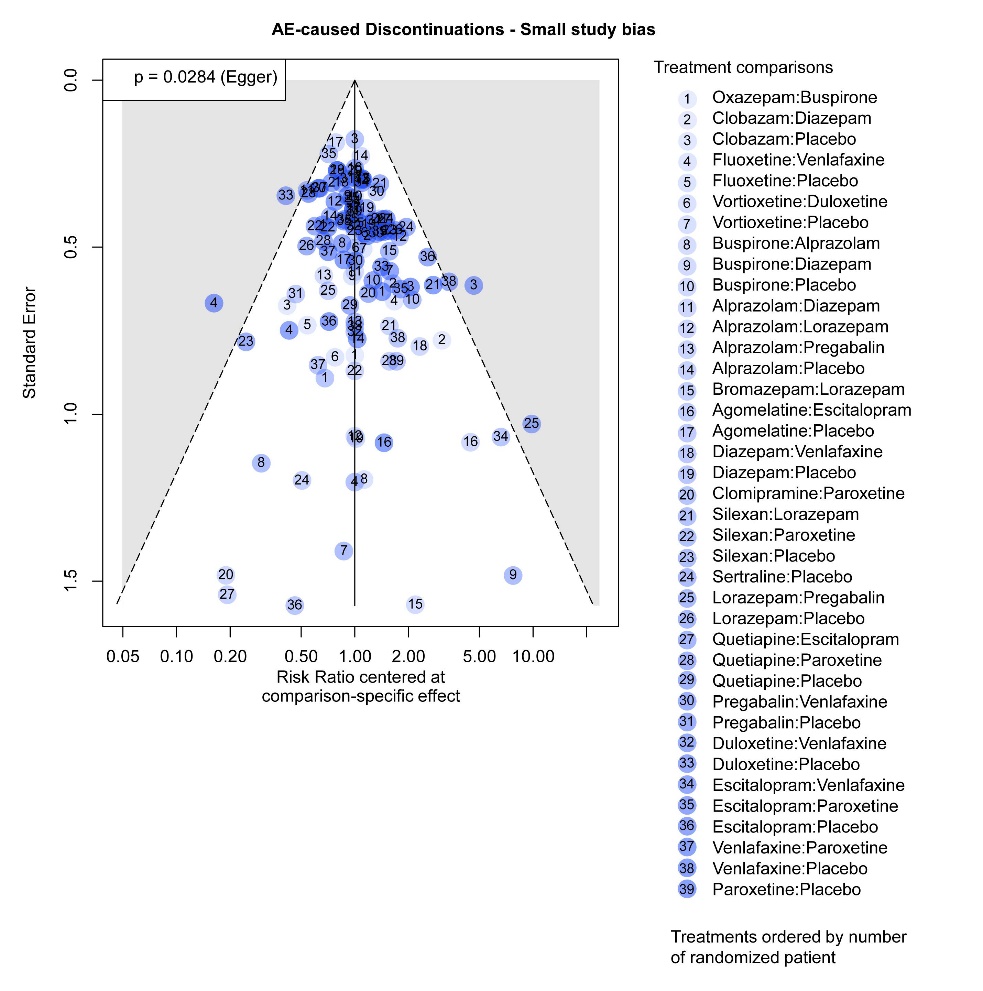** |
| **f) AE-caused discontinuations (zoomed in)**  **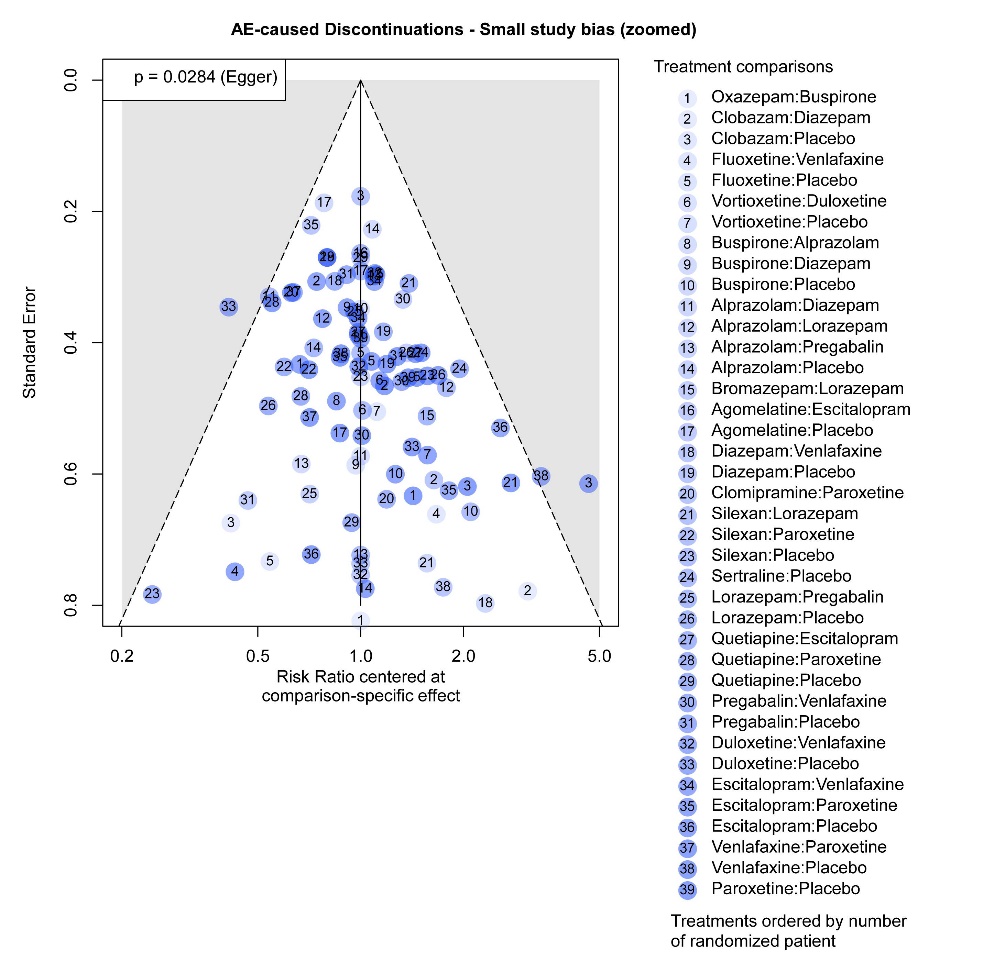** |
| **g) AEs**  **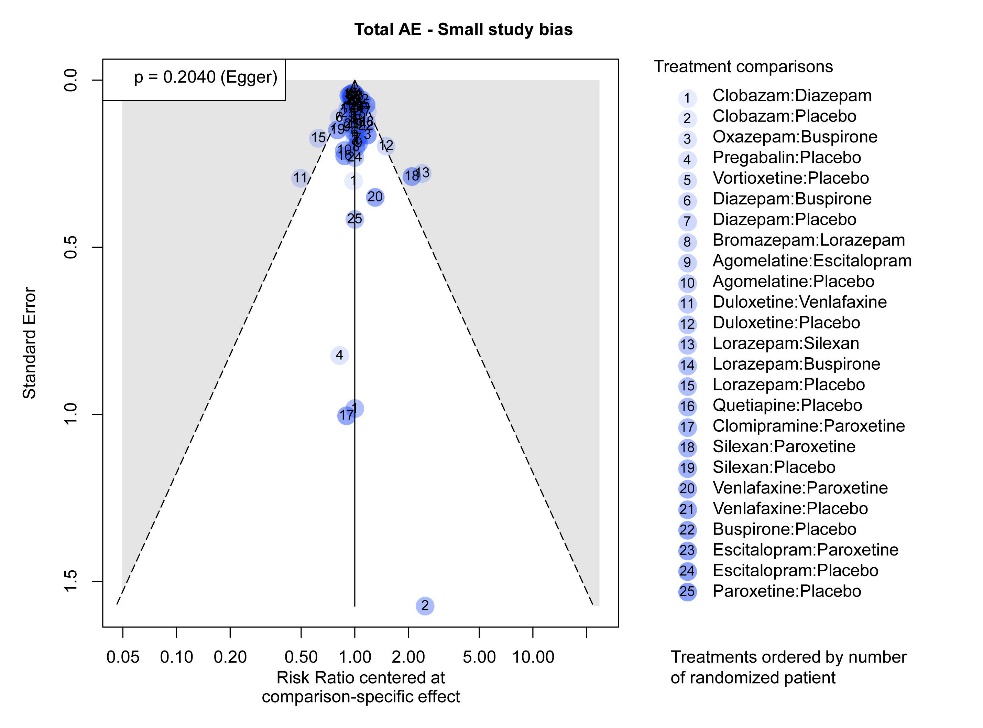** |
| **h) AEs (zoomed in)**  **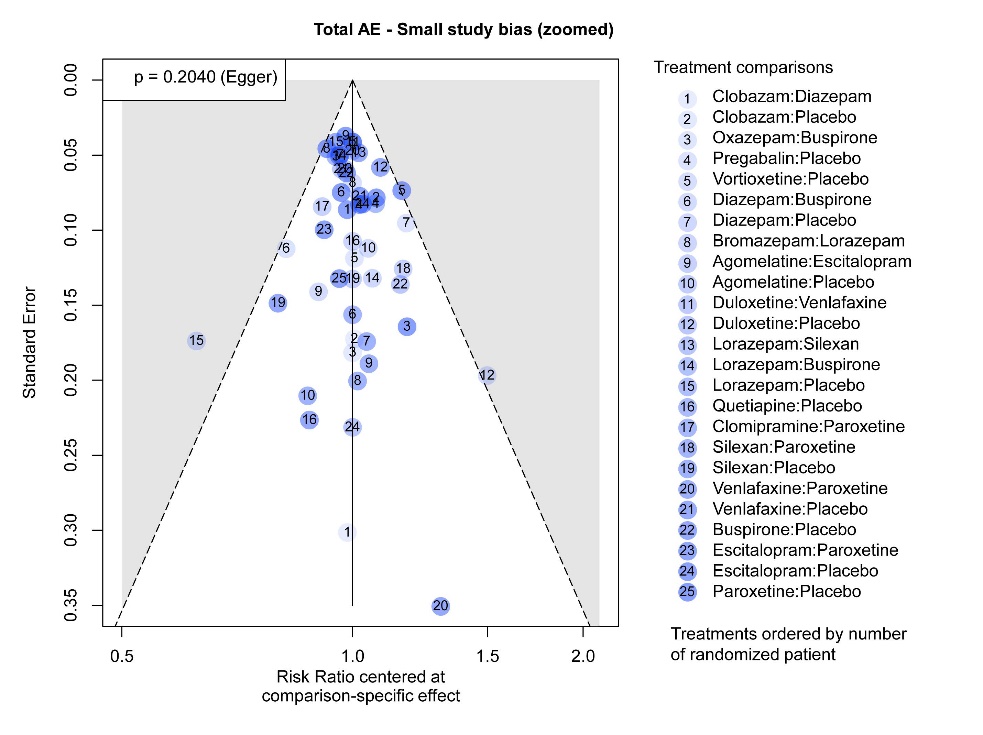** |
| **i) Light AEs**  **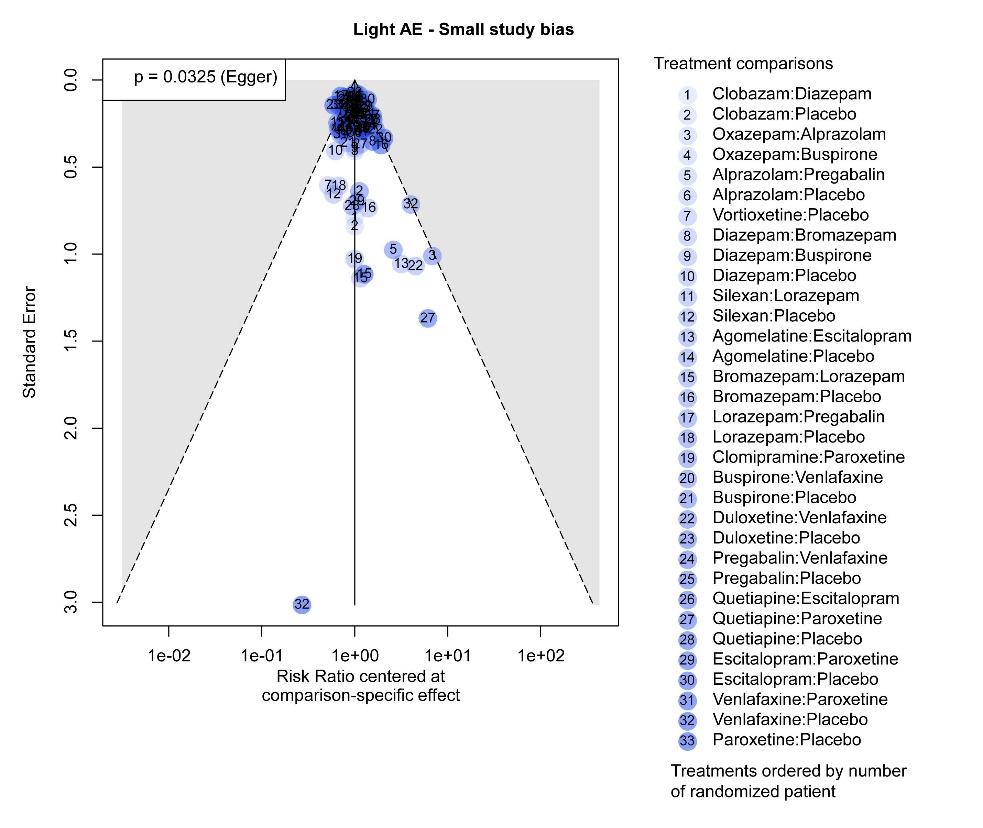** |
| **j) Light AEs (zoomed in)**  **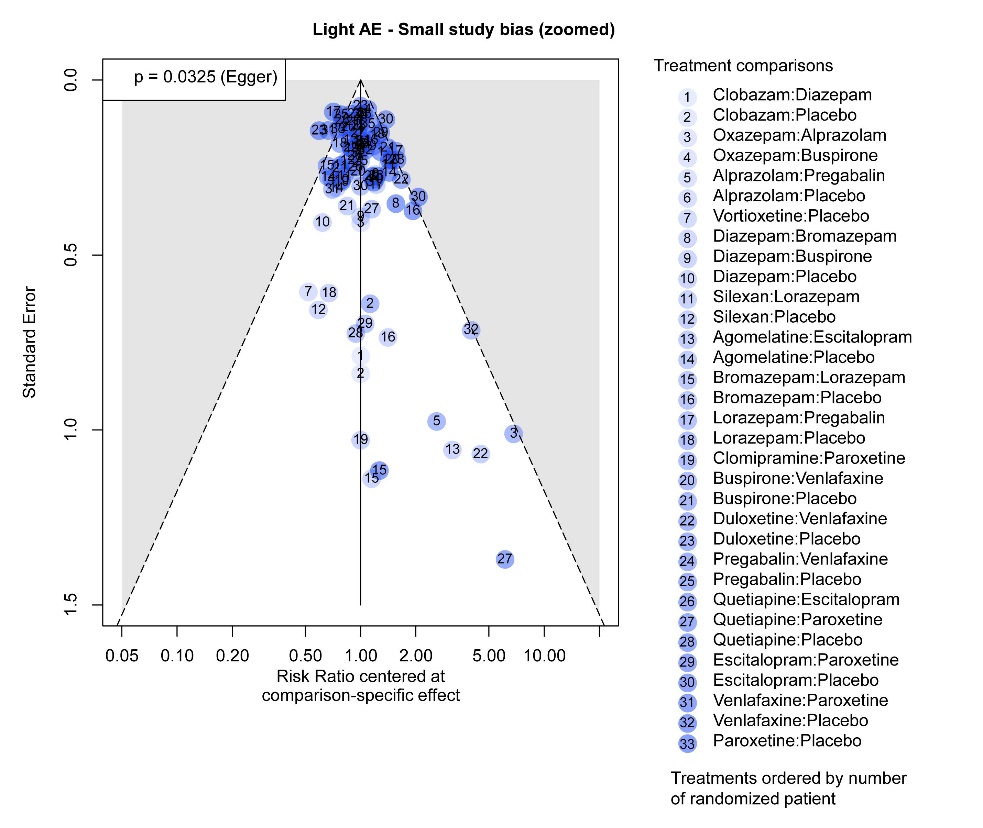** |
| **k) Moderate AEs**  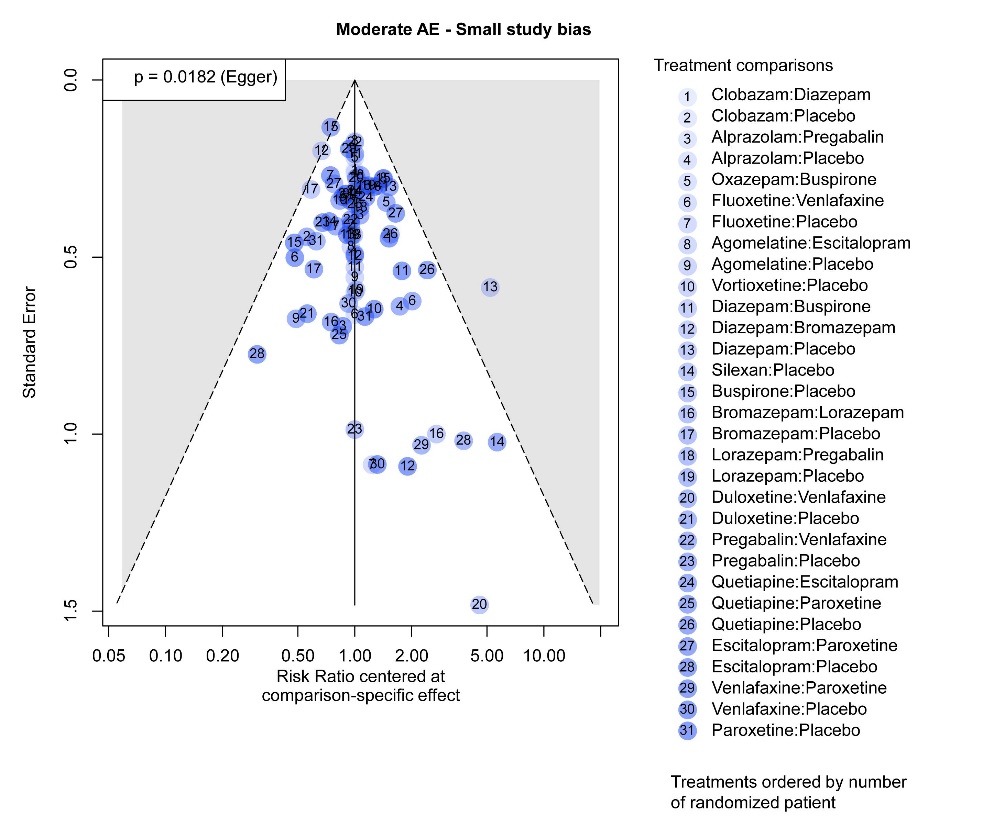 |
| **l) Moderate AEs (zoomed in)**  **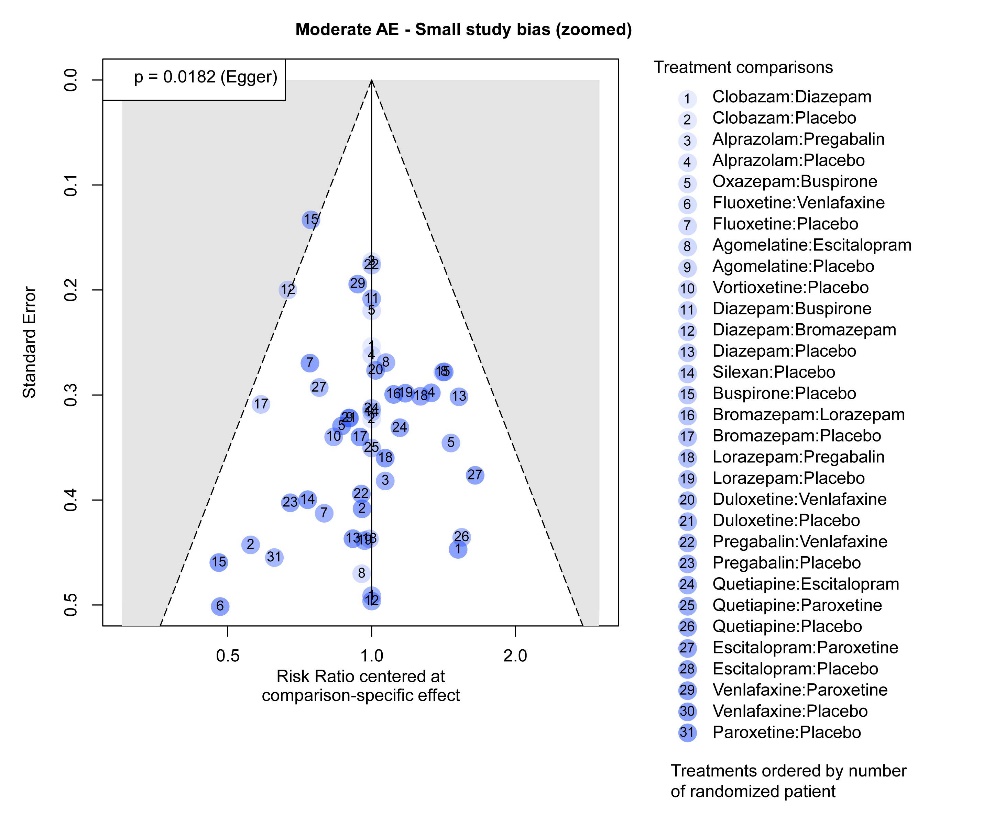** |

AEs, adverse events; HAM-A, Hamilton Anxiety Rating Scale.

**Figures S3. Funnel plots – publication time bias (ordered by median publication date)**

| **a) HAM-A**  **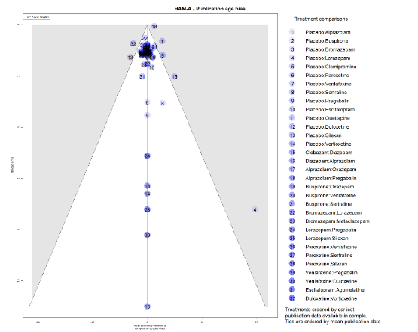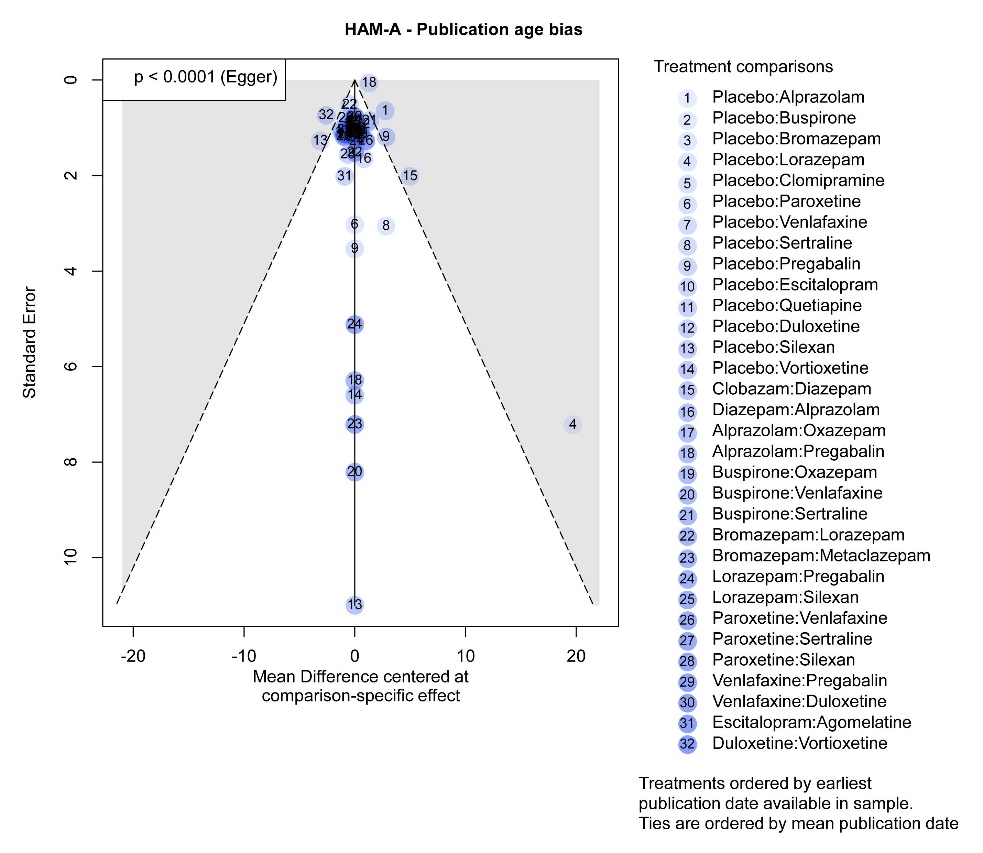** |
| --- |
| **b) HAM-A (zoomed in)**  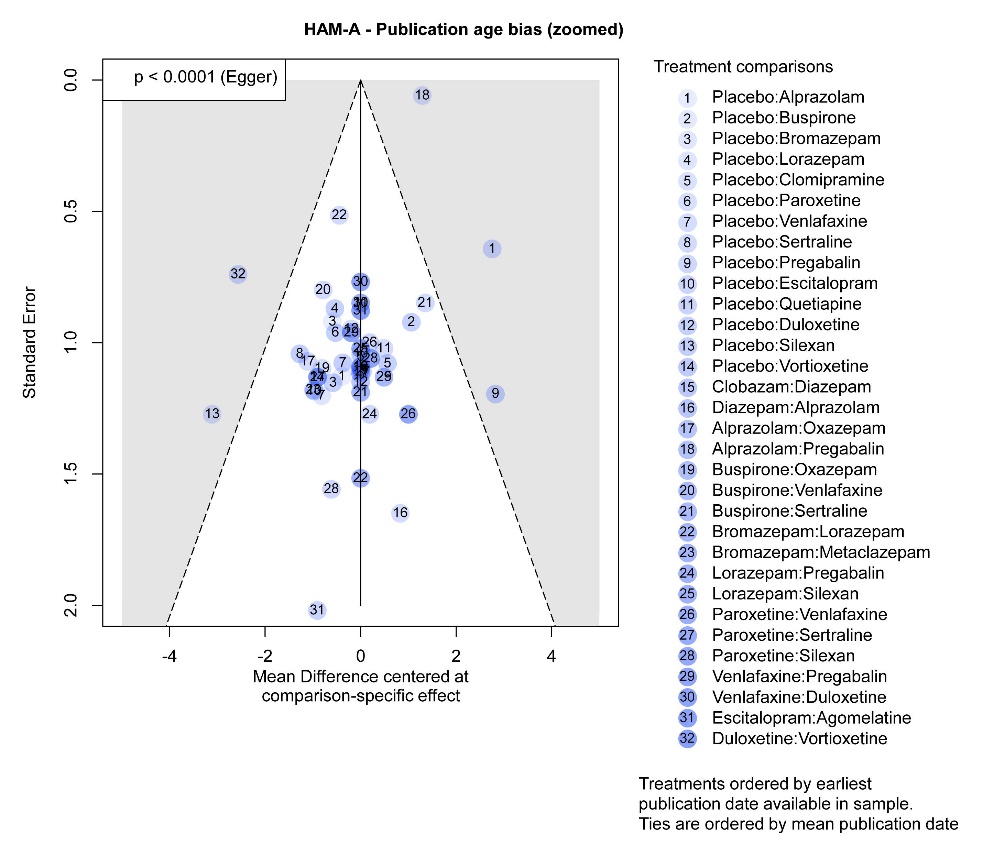 |
| **c) All-cause discontinuations**  **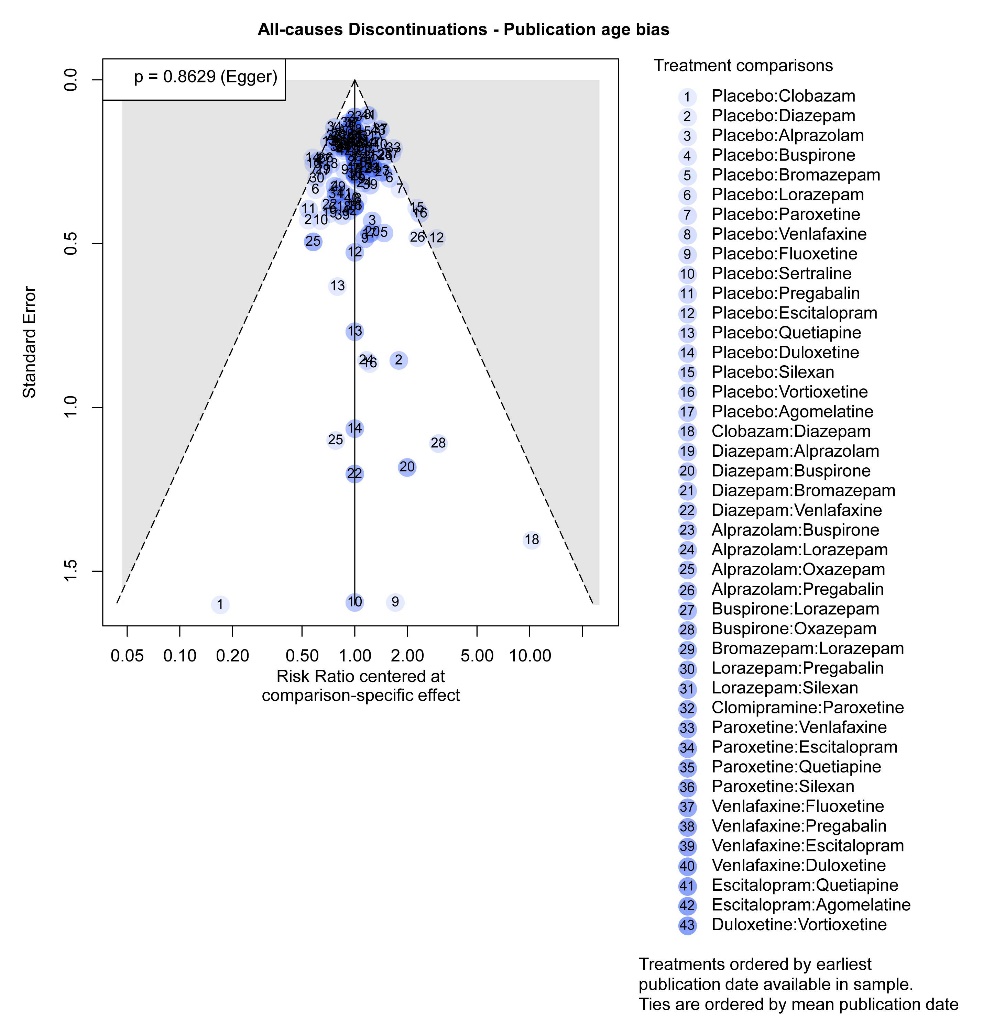** |
| **d) All-cause discontinuations (zoomed in)**  **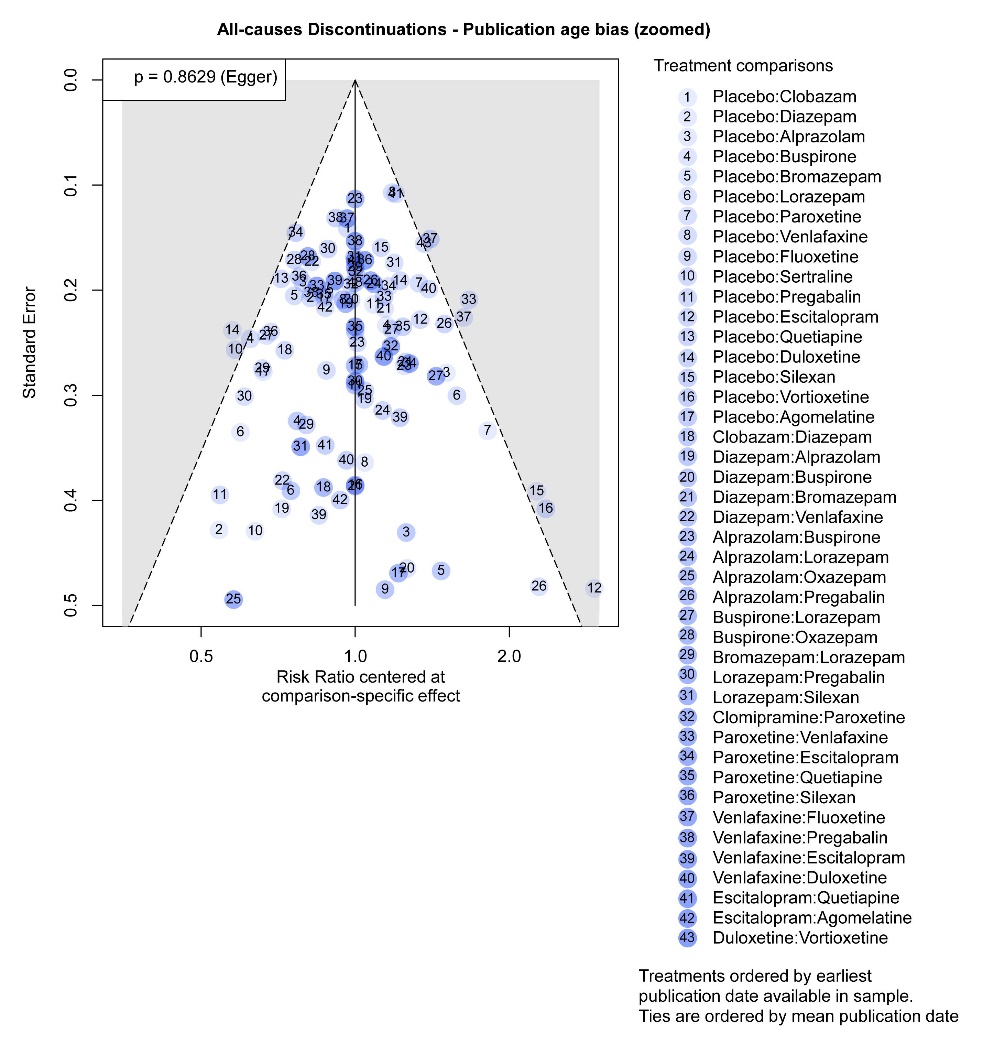** |
| **e) AE-caused discontinuations**  **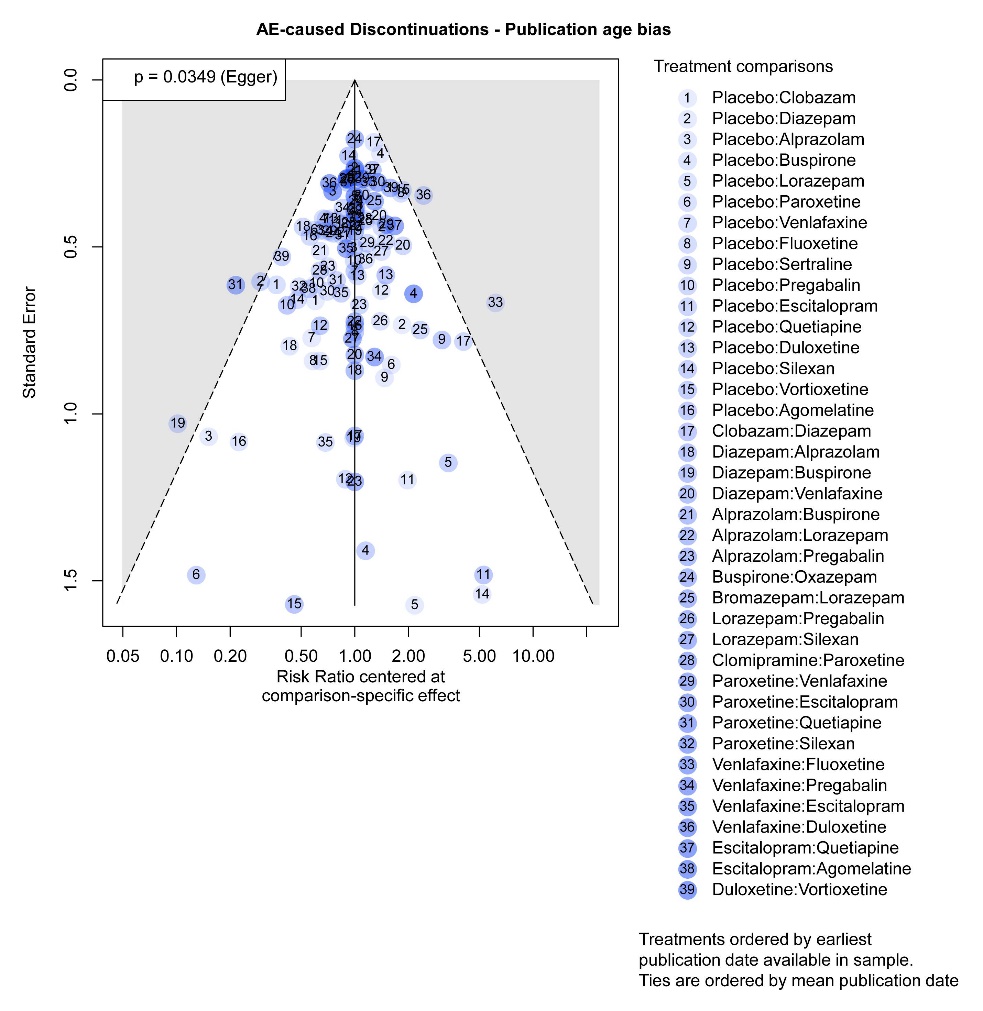** |
| **f) AE-caused discontinuations (zoomed in)**  **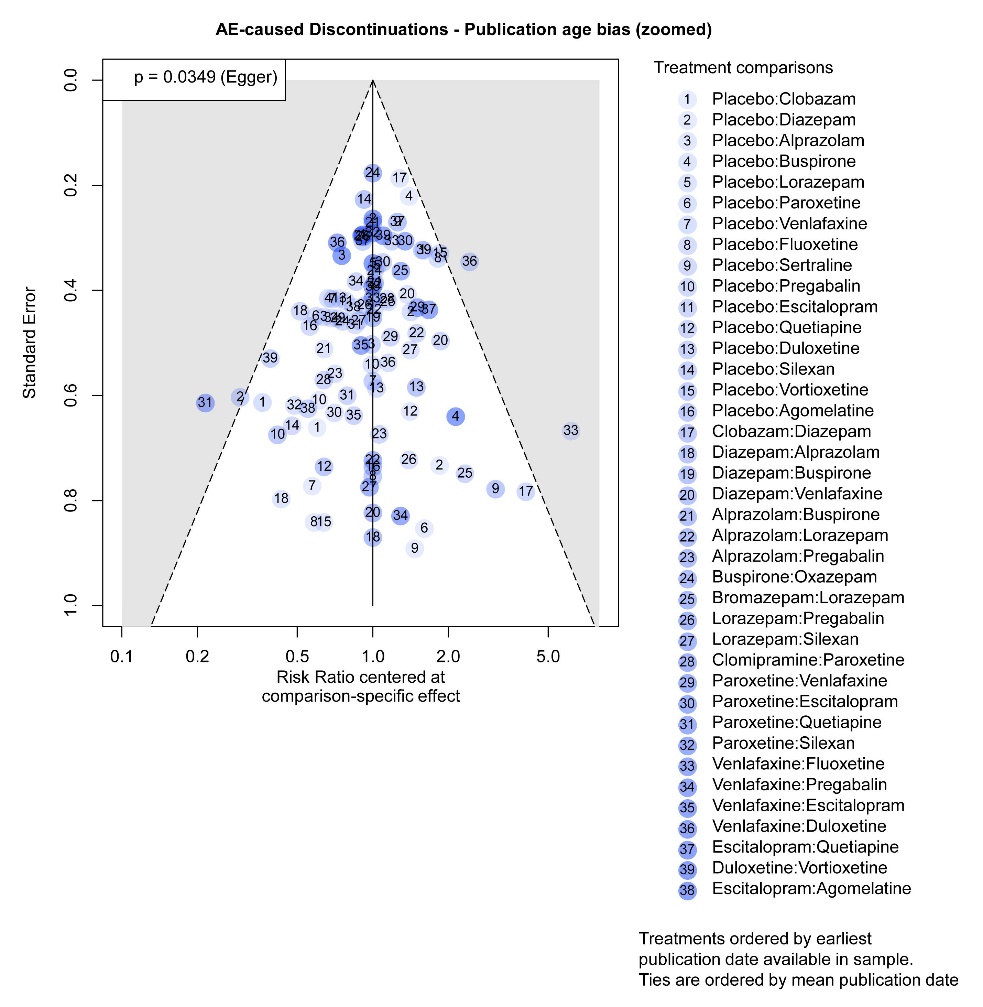** |
| **g) AEs**  **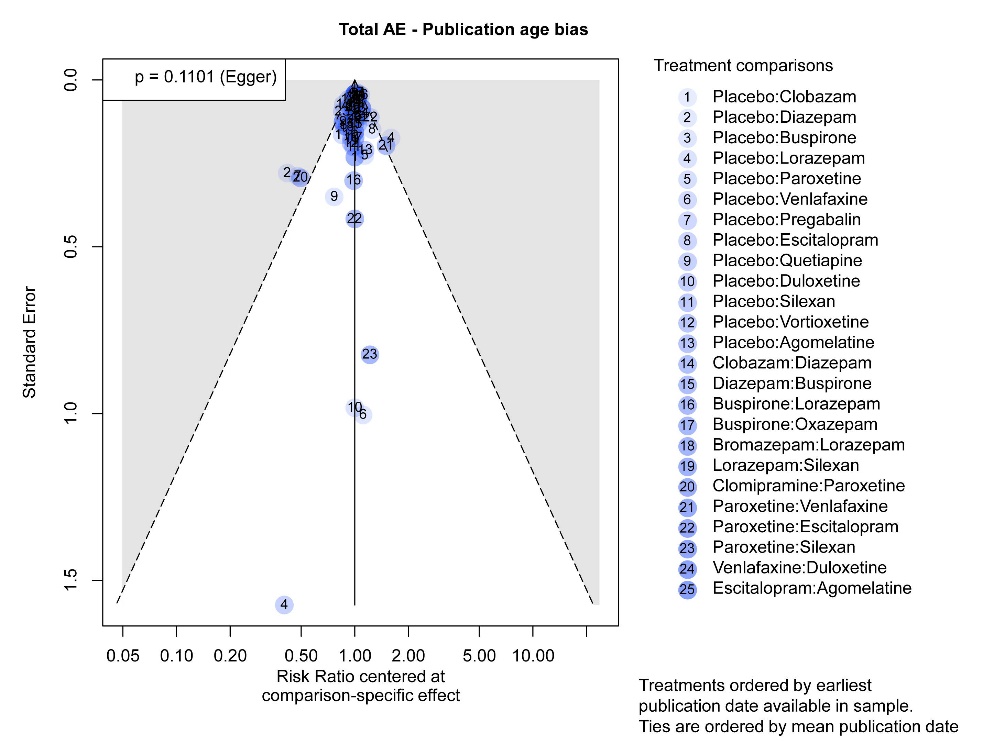** |
| **h) AEs (zoomed in)**  **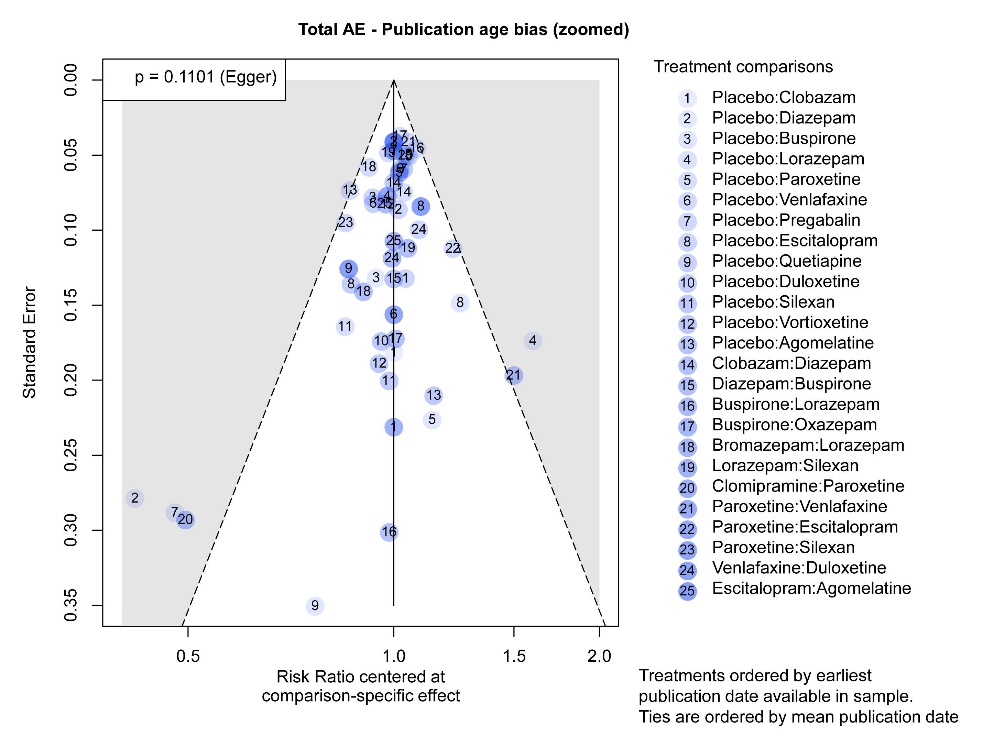** |
| **i) Light AEs**  **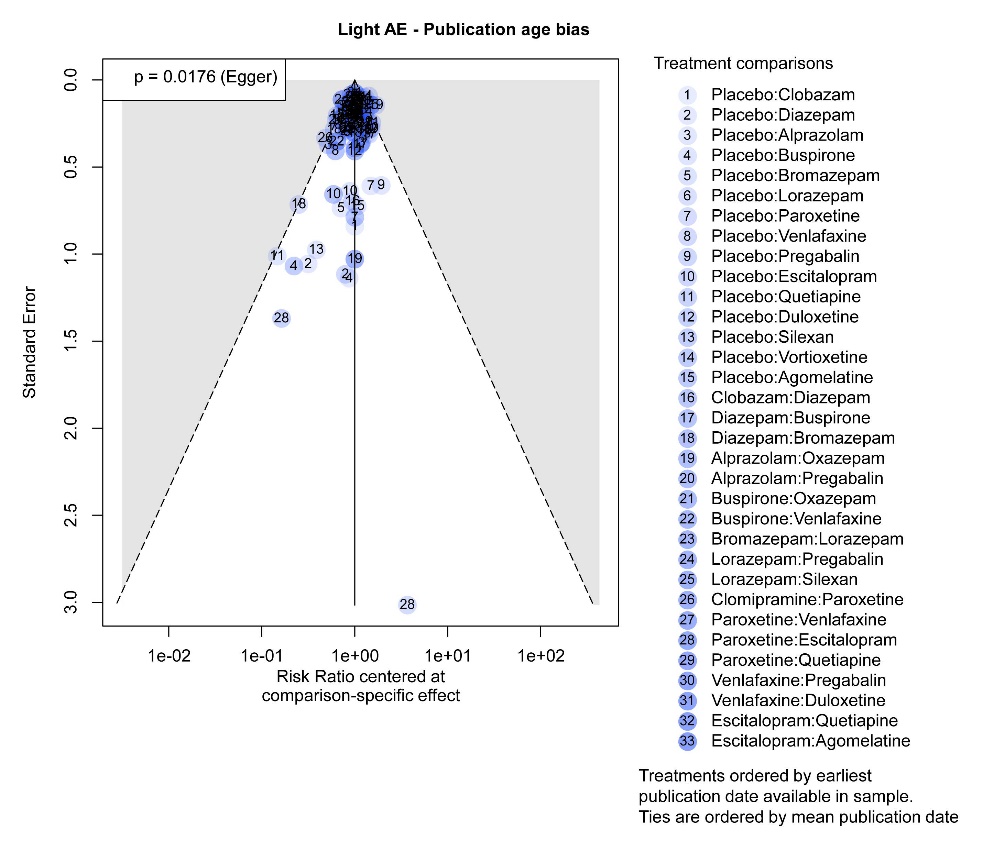** |
| **j) Light AEs (zoomed in)**  **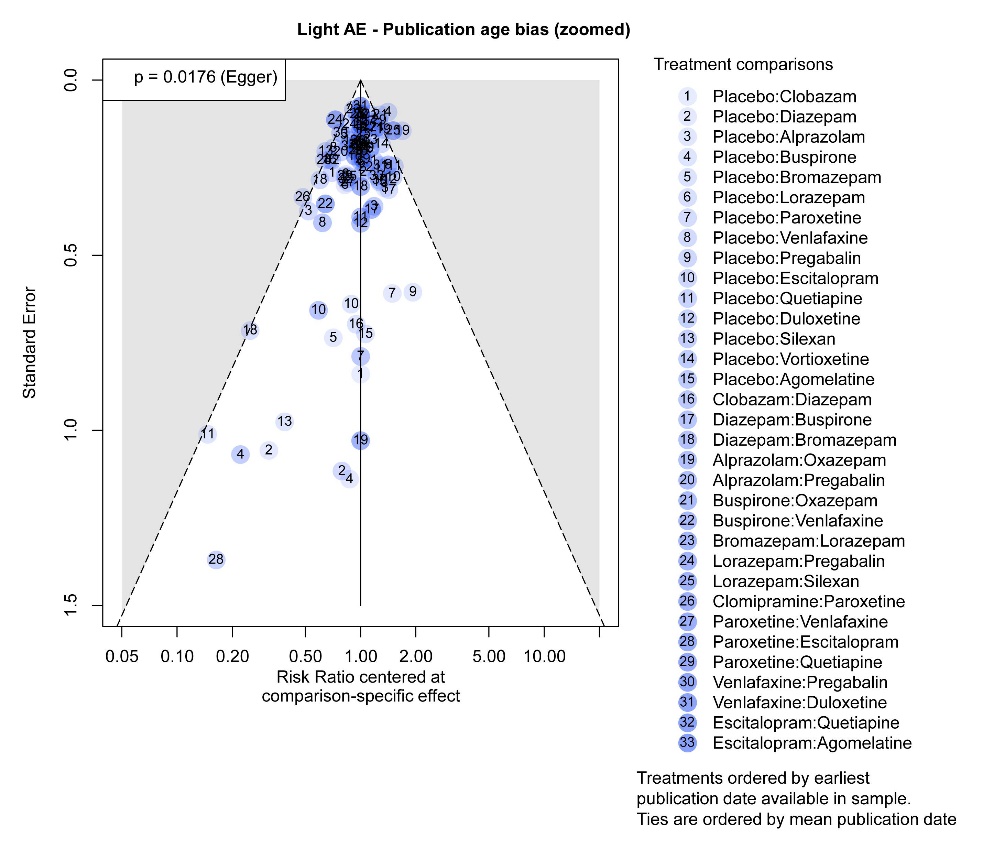** |
| **k) Moderate AEs**  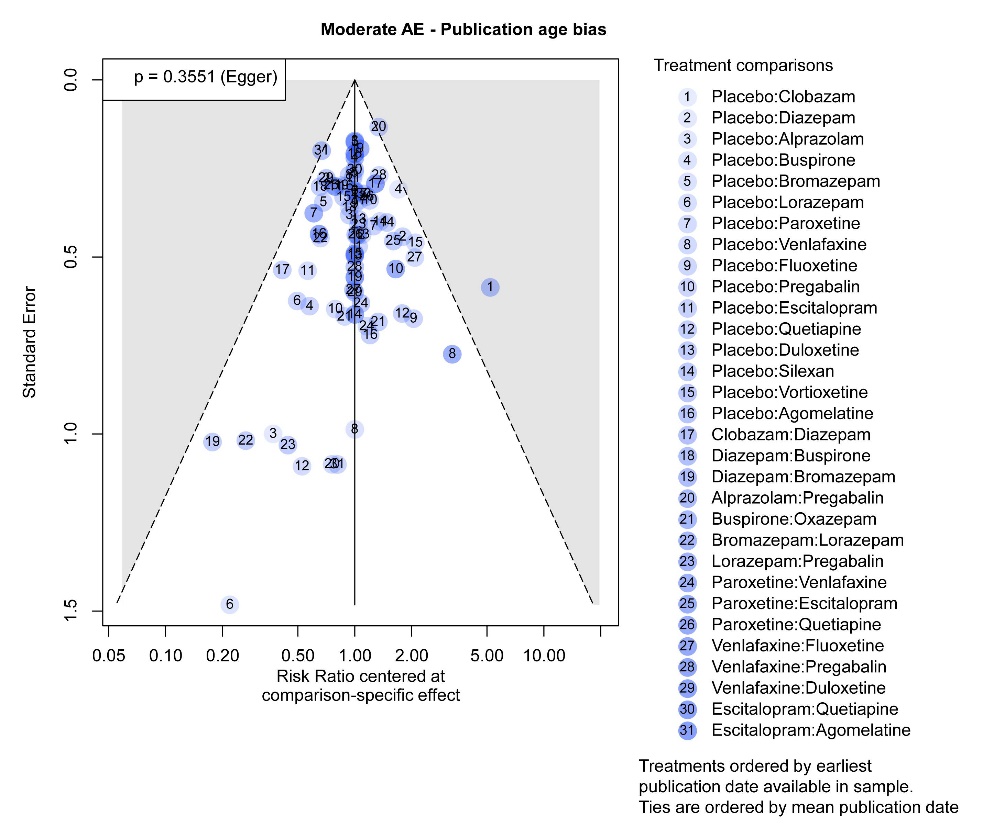 |
| **l) Moderate AEs (zoomed in)**  **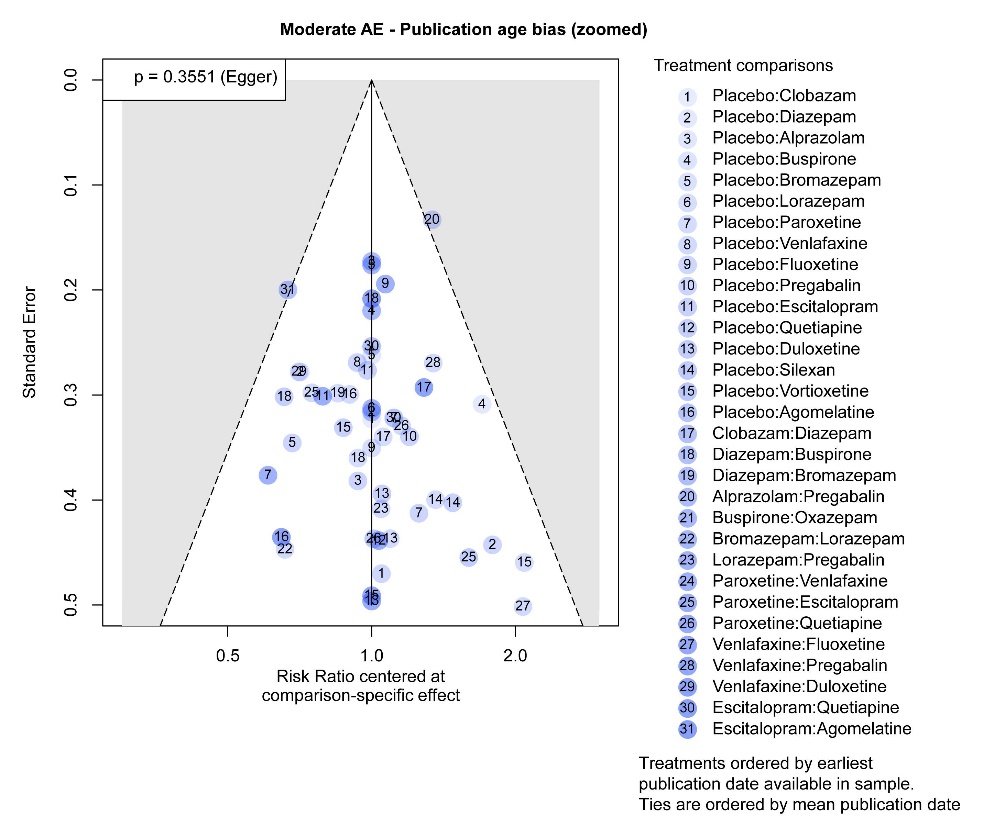** |

AEs, adverse events; HAM-A, Hamilton Anxiety Rating Scale.
